# Paule‐Mandel estimators for network meta‐analysis with random inconsistency effects

**DOI:** 10.1002/jrsm.1244

**Published:** 2017-06-05

**Authors:** Dan Jackson, Areti Angeliki Veroniki, Martin Law, Andrea C. Tricco, Rose Baker

**Affiliations:** ^1^ MRC Biostatistics Unit Cambridge UK; ^2^ Li Ka Shing Knowledge Institute Toronto Canada; ^3^ Epidemiology Division, Dalla Lana School of Public Health University of Toronto Toronto Canada; ^4^ University of Salford Manchester UK

**Keywords:** incoherence, mixed treatment comparisons, multiple treatments meta-analysis, random-effects models

## Abstract

Network meta‐analysis is used to simultaneously compare multiple treatments in a single analysis. However, network meta‐analyses may exhibit inconsistency, where direct and different forms of indirect evidence are not in agreement with each other, even after allowing for between‐study heterogeneity. Models for network meta‐analysis with random inconsistency effects have the dual aim of allowing for inconsistencies and estimating average treatment effects across the whole network. To date, two classical estimation methods for fitting this type of model have been developed: a method of moments that extends DerSimonian and Laird's univariate method and maximum likelihood estimation. However, the Paule and Mandel estimator is another recommended classical estimation method for univariate meta‐analysis. In this paper, we extend the Paule and Mandel method so that it can be used to fit models for network meta‐analysis with random inconsistency effects. We apply all three estimation methods to a variety of examples that have been used previously and we also examine a challenging new dataset that is highly heterogenous. We perform a simulation study based on this new example. We find that the proposed Paule and Mandel method performs satisfactorily and generally better than the previously proposed method of moments because it provides more accurate inferences. Furthermore, the Paule and Mandel method possesses some advantages over likelihood‐based methods because it is both semiparametric and requires no convergence diagnostics. Although restricted maximum likelihood estimation remains the gold standard, the proposed methodology is a fully viable alternative to this and other estimation methods.

## 1 INTRODUCTION

Meta‐analysis is now well established. Simple and transparent univariate methods are typically used in systematic reviews and meta‐analyses. However more sophisticated methodologies have been suggested in recent years and network meta‐analysis^1^, ^2^ is one such development that has attracted considerable interest. Here, data from multiple (more than 2) treatment groups are included in a single analysis. Network meta‐analyses allow both direct and indirect evidence for multiple treatment groups to contribute to the analysis so that more precise and coherent inferences about all possible treatment comparisons are possible. Models for network meta‐analysis may include random‐effects to account for any between‐study heterogeneity so that the usual univariate random‐effects model for univariate meta‐analysis^3^, ^4^, ^5^, ^6^ can be extended to the network meta‐analysis setting.^7^ Assuming that there is no between‐study heterogeneity is usually difficult to defend in both univariate and network meta‐analysis.

Network meta‐analysis raises additional concerns about whether or not the various sources of evidence in the network are consistent. We will take the consistency assumption to mean that for example, to within‐study statistical error and between‐study variation, that the relative effect of treatment *B* to treatment *A*, plus the effect of treatment *C* to treatment *B*, equals the relative effect of treatment *C* to treatment *A*. This assumption means that the direct evidence for the *AC* comparison is in agreement with the indirect evidence” (“*AB* + *BC* = *AC*”). Estimated treatment effects that come from the same study will satisfy this type of consistency equality without error. This is because of the way that relative effects are calculated.

A direct way to model inconsistency is to allow studies that include different combinations of treatment comparisons to estimate different effects. If this occurs then any closed loops, such as the *ABC* loop discussed above, will not “add up correctly”: if studies that include treatments *A* and *B* (“*AB* studies”) estimate different effects to *BC* and *AC* studies, then the consistency assumption will not hold. Explaining inconsistency in this way is especially natural because different treatment comparisons may have been made at slightly different times, using different types of patients or in other circumstances that affect the treatment outcome. This may either be known, suspected, or unknown to the analyst. The inconsistency modelling follows this intuition, by allowing studies of each “design” to estimate different effects (where “design” is taken only to refer to the treatments included). This results in the design‐by‐treatment interaction model.^8^, ^9^ This type of modelling has also been proposed by Piepho and colleagues.^10^, ^11^ By allowing a full interaction between the treatment effects and the type of design we avoid the problems associated with loop‐inconsistency models when multiarm studies are present.^8^, ^12^ Briefly, the difficulty with loop‐inconsistency models is that the form of the model depends on the treatment ordering when multi‐arm studies are present. The relationship between loop inconsistency models and the design‐by‐treatment interaction model is briefly described below, and see Higgins et al^8^ and Jackson et al^12^ for full details.

We will adopt a relatively simple form of the design‐by‐treatment interaction model that uses two unknown variance parameters and has been described previously.^13^, ^14^, ^15^ One of these variance parameters describes the extent of the between‐study heterogeneity. The other describes the extent of inconsistency, using a simple model eases model identifiability and interpretation. Using random, rather than fixed, inconsistency parameters means that the basic parameters describe the average treatment effects across studies of all designs. Hence, the decision to use random inconsistency effects is motivated by the dual desire to include the possibility of inconsistency in the model and estimate meaningful average treatment effects across the entire evidence base.

Bayesian estimation methods for our model have previously been suggested^13^, ^15^ and two classical estimation methods have also been developed. The first of these classical methods uses the method of moments^14^ and extends the univariate method proposed by DerSimonian and Laird.^4^ The second instead uses likelihood‐based methods.^15^ The primary contribution of this paper is to develop a third such estimation method, this time extending the univariate method proposed by Paule and Mandel.^16^ See Veroniki et al^17^ for details of this and many other estimation methods that have been proposed in the more familiar pairwise meta‐analysis setting.

The Paule and Mandel estimation method retains many of the advantages of the method of moments because it is semiparametric and requires no convergence diagnostics. Furthermore, it has not been criticised for being inaccurate in the way that the DerSimonian and Laird method has (eg, Hoaglin^18^ and Kulinskaya et al^19^). Rukhin et al^20^ show that the Paule and Mandel estimator is an approximate version of using restricted maximum likelihood (REML). Viechtbauer et al^21^ show that the Paule and Mandel estimator is the same as the so‐called empirical Bayes estimator. Findings such as these strengthen the case for using the Paule and Mandel estimator. Although REML might reasonably be regarded as the gold standard, this is a fully parametric estimation method, whereas the Paule and Mandel and DerSimonian and Laird methods allow us to estimate the unknown between‐study variance without making normality assumptions. However, the extent to which this is truly an advantage of using semiparametric methods awaits investigation and is debatable, because we can anticipate that likelihood based methods for network meta‐analysis will be robust to departures from non‐normality, just as they have been found to be in the univariate setting.^22^, ^23^ Furthermore both the likelihood and the restricted likelihood must be maximised numerically and convergence should be checked in practice. As we will see below, the Paule and Mandel method requires no such convergence diagnostics. The new Paule and Mandel method that we develop for network meta‐analysis therefore possesses some advantages over the other two classical methods.

The rest of the paper is set out as follows. In Section 2, we describe the model, and in Section 3, we discuss the previously proposed estimation methods. In Section 4, we present the new Paule and Mandel estimators for network meta‐analysis. In Section 5, we apply all 3 classical methods to some examples that have been used previously to illustrate these methods. We also apply these methods to a challenging new example in this section. In Section 6, we perform a simulation study and we conclude with a short discussion in Section 7.

## 2 THE MODEL

Our model has been described previously,^13^, ^14^, ^15^ but we also present it here. We model the estimated treatment effects using the equation

(1) $$Y_{\mathrm{di}}=\delta_{d}+B_{\mathrm{di}}+\omega_{d}+\varepsilon_{\mathrm{di}}$$

where **Y**_*di*_ represents the *c*_*d*_ × 1 vector of estimated treatment effects from the *i*th study of design *d* = 1 , 2 ,  ⋯  , *D*, where *c*_*d*_ is the number of treatment arms in design *d* minus one. The **Y**_*di*_ are the estimated relative effects of different treatment comparisons (such as log odds ratios or mean differences). We therefore use contrast based models and analyses throughout. An arm‐based analysis would instead model the average outcome in each treatment arm (such as log odds or the sample mean). The arguments for and against these contrasting types of model are sometimes fierce^24^, ^25^ but by adopting a contrast based approach we use the more conventional approach.^24^ The ***ε***_*di*_ are the within‐study statistical errors, and we assume that ***ε***_*di*_ ~ *N*(**0**, **S**_*di*_), where **S**_*di*_ is the *c*_*d*_ × *c*_*d*_ within‐study covariance matrix. The within‐study covariance matrix is estimated in practice but is treated as fixed and known in the analysis. Hence, we will use the conventional type of normal approximation that meta‐analysts will be familiar with. The terms **B**_*di*_ and ***ω***_*d*_ in model (1) are random effects that describe the between‐study heterogeneity and the inconsistency, respectively, and are described in more detail below.

### 2.1 Calculating meta‐analysis data

To calculate the outcome data **Y**_*di*_ in model (1), we choose a design‐specific baseline treatment group (eg, *A* in the *ABC* design). The entries of **Y**_*di*_ are then obtained as the estimated treatment effects of the other *c*_*d*_ treatments compared to this baseline treatment. For example if *d* = 1 is taken to indicate the *ABC* design then the **Y**_1*i*_ vectors have two entries. If *A* is taken as the baseline then these two entries of the **Y**_1*i*_ are estimated treatment effects of *B* and *C* relative to *A*. The main diagonal entries of the **S**_*di*_ are within‐study variances that can be calculated using standard methods. The within‐study correlations in the estimated effects from multiarm studies are because they share a common baseline treatment. Hence the within‐study covariances, the off‐main diagonal entries of **S**_*di*_, are the variance of the average outcome (for example, the log odds or the sample mean) in the baseline treatment group. For designs that include only two treatment groups, both **Y**_*di*_ and **S**_*di*_ are scalars. Forming the outcome data **Y**_*di*_ and the within‐study covariance matrices **S**_*di*_ to be used in analysis is only slightly more complicated for network meta‐analysis than conventional univariate analyses.

### 2.2 The basic parameters

We choose a reference treatment *A* for the entire network meta‐analysis and we denote the average (ie, across all designs and studies) treatment effects relative to *A* as *δ*^*AB*^, *δ*^*AC*^, and so on, where we refer to these as “basic parameters.” There are *c* of these basic parameters, where *c* is the number of treatment groups minus one. Then the fixed effect ***δ***_*d*_ in model (1) represents the average treatment effects of design *d* in terms of these basic parameters. For designs that include the reference treatment *A*, the entries of ***δ***_*d*_ are simply given by the basic parameter that corresponds to the appropriate treatment comparison; for example continuing with the above example ***δ***_1_ = (*δ*^*AB*^, *δ*^*AC*^)^*T*^. For designs that do not include the reference treatment *A*, forming ***δ***_*d*_ is a little more complicated. For example, if *d* = 2 indicates the *CDE* design we may take treatment *C* as the design‐specific baseline so that the entries of the **Y**_2*i*_ are the estimated treatment effects of *D* and *E* relative to *C*. Then ***δ***_2_ = (*δ*^*AD*^ − *δ*^*AC*^, *δ*^*AE*^ − *δ*^*AC*^)^*T*^. In general, the average effect of treatment *J* relative to treatment *I* is *δ*^*AJ*^ − *δ*^*AI*^, where we define *δ*^*AA*^ = 0.

### 2.3 The random‐effects

The **B**_*di*_ are random‐effects that describe the between‐study heterogeneity and the ***ω***_*d*_ are random‐effects that describe the inconsistency. The inclusion of the ***ω***_*d*_ in model (1) means that every design estimates a different set of effects. Hence, we can describe the inconsistency in the way described in the introduction. The simplest possible model in our framework takes **B**_*di*_ = ***ω***_*d*_ = **0**. There is then no between‐study heterogeneity or inconsistency. We call this the “common‐effect and consistent model.”

We assume that $B_{\mathrm{di}}\sim N\left(0,\tau_{\beta }^{2}P_{c_{d}}\right)$, where $P_{c_{d}}$ is a square matrix of dimension *c*_*d*_ with ones on the main diagonal and halves everywhere else. Similarly, we assume that $\omega_{d}\sim N\left(0,\tau_{\omega }^{2}P_{c_{d}}\right)$. The unknown variances $\tau_{\beta }^{2}$ and $\tau_{\omega }^{2}$ describe the magnitude of the between‐study, and the inconsistency, variance, respectively. These two parameters can therefore be referred to as the between‐study variance and the inconsistency variance. These are very simple models for the between‐study heterogeneity and the inconsistency structure, which assume that the between‐study and inconsistency variances are the same for all studies and designs, respectively, regardless of the particular treatments being compared.^13^, ^14^, ^15^ If $\tau_{\beta }^{2}=0$ then all **B**_*di*_ = **0** and there is no between‐study heterogeneity, similarly, if $\tau_{\omega }^{2}=0$, then all ***ω***_*d*_ = **0** and the data are consistent. If $\tau_{\omega }^{2}>0$ then the estimates from studies of the same design are correlated because they share a common inconsistency parameter. These assumptions mean that the covariance matrix of **Y**_*di*_ is $\left(\tau_{\beta }^{2}+\tau_{\omega }^{2}\right)P_{c_{d}}+S_{\mathrm{di}}$ and the covariance between **Y**_*di*_ and **Y**_*dj*_ of the same design is $\tau_{\omega }^{2}P_{c_{d}}$.

### 2.4 Describing the entire dataset

Model (1) describes the estimated effects for each study separately. To describe the entire dataset, we vertically stack the **Y**_*di*_ to create **Y**, where model (1) implies that

(2) $$Y\sim N\left(X\delta ,\tau_{\beta }^{2}M_{1}+\tau_{\omega }^{2}M_{2}+S\right),$$

where ***δ*** is a vector that contains the basic parameters. This vector is premultiplied by design matrix **X**, where the design matrix ensures that model (2) provides the mean structure implied by model (1). The block diagonal matrix **S** contains the **S**_*di*_ matrices along the main block diagonal, so that the within‐study distributions in models (1) and (2) are equivalent. Similarly, the terms $\tau_{\beta }^{2}M_{1}$ and $\tau_{\omega }^{2}M_{2}$ ensure the equivalence between the between‐study and inconsistency variance structures, respectively, in models (1) and (2). This is achieved by defining square matrices **M**_1_ and **M**_2_ so that the covariance structure from Equation (1) is correctly specified in Equation (2). Specifically, **M**_1_ contains ones on the main diagonal, so that **M**_1*ii*_ = 1 for all *i*. Furthermore, for *i* ≠ *j*, **M**_1*ij*_ = 1/2 if the corresponding entries (ie, rows) of **Y** are from the same study, and **M**_1*ij*_ = 0 otherwise. We similarly define **M**_2_ as containing ones on the main diagonal so that **M**_2*ii*_ = 1 for all *i*. Furthermore, for *i* ≠ *j*, **M**_2*ij*_ = 1 if the corresponding entries of **Y** are from the same design and refer to the same treatment comparison, **M**_2*ij*_ = 1/2 if they are from the same design but refer to different treatment comparisons, and **M**_2*ij*_ = 0 otherwise. Matrix **M**_1_ is a block‐diagonal square matrix with blocks formed by the studies and **M**_2_ a block‐diagonal square matrix with blocks formed by the designs.

The matrices **M**_1_ and **M**_2_ were referred to as **P**_1_ and **P**_2_ in the first description of this model,^14^ but in the recent account,^15^ we use the letter **M** to denote these matrices to avoid a clash of notation with $P_{c_{d}}$. Matrices **M**_1_ and **M**_2_ are both symmetrical matrices; this observation is important when deriving some of the results that follow.

### 2.5 Loop inconsistency models and the consistency model

Loop inconsistency models^7^ are a special case of the design‐by‐treatment interaction model.^8^, ^12^ This means that loop inconsistency models can be assumed by modifying **M**_2_. 14 All estimation methods below can similarly be modified to accommodate this type of model by using the appropriate **M**_2_ matrix. We can also assume a consistency model by taking $\tau_{\omega }^{2}=0$. Our proposal is to fit our full model but these and possibly other reduced forms of our model may appeal to some readers.

### 2.6 A submodel for a single design

Jackson et al^14^ describe a submodel of Equation (2) that can be used to describe outcome data from a particular design. Only attempting to describe the outcome data from one design simplifies matters. This is because inconsistency is conceptualised as being differences between designs so that the inconsistency (or “between‐designs”) variance $\tau_{\omega }^{2}$ is not identifiable unless two or more designs are modelled. This also means that the **Y**_*di*_ from a particular design are consistent with each other and from Equation (1) estimate ***β***_*d*_ = ***δ***_*d*_ + ***ω***_*d*_. All entries of ***δ*** will not in general be identifiable when using data from a single design because we will not observe all treatments unless the design in question includes them all. Finally, the vectors ***δ***_*d*_ and ***ω***_*d*_ are aliased in model (1) when all studies are of the same design, so we cannot identify ***δ***_*d*_ and ***ω***_*d*_ separately but we can identify their sum ***β***_*d*_.

Hence, a submodel, which is implied by model (2) and can be identified when all studies are of a single design *d*, is

(3) $$Y_{d}\sim N\left(X_{d}\beta_{d},\tau_{\beta }^{2}M_{d}+S_{d}\right),$$

where **Y**_*d*_ is obtained by stacking the **Y**_*di*_ that are from design *d* and $M_{d}=I_{n_{d}}⊗P_{c_{d}}$; here, *n*_*d*_ is the number of studies of design *d*, $I_{n_{d}}$ denotes the *n*_*d*_ × *n*_*d*_ identity matrix, and ⊗ denotes the Kronecker product. Hence, $M_{d}=I_{n_{d}}⊗P_{c_{d}}$ is a block diagonal square matrix where all submatrices along the block diagonal are $P_{c_{d}}$. The design matrix **X**_*d*_ is obtained by stacking *n*_*d*_ identity matrices of dimension *c*_*d*_. Finally **S**_*d*_ is the block diagonal square matrix containing the **S**_*di*_ matrices for the design in question. The matrix **M**_*d*_ is symmetrical.

Submodel (3) states that all **Y**_*di*_ from design *d* independently estimate *β*_*d*_. There is therefore no inconsistency in these estimates in this submodel because Equation (3) includes no inconsistency effects or variances. Submodel (3) is implied by model (2) regardless of the value of $\tau_{\omega }^{2}$ and would also be implied if the ***ω***_*d*_ were modelled differently, for example, using a different random‐effects distribution or modelled using fixed‐effects.

### 2.7 Making inferences about the treatment effects

The main statistical difficulty lies in estimating the unknown variance components $\tau_{\beta }^{2}$ and $\tau_{\omega }^{2}$. Once these parameters have been estimated, the standard procedure for making inferences about the basic parameters, and so the treatment effects, approximates these variance parameters with their estimates. Inference for the basic parameters is then straightforward, as analysis proceeds as a weighted regression where all weights are treated as fixed and known.^14^, ^15^ Hence the estimation of $\tau_{\beta }^{2}$ and $\tau_{\omega }^{2}$ provides the focus of the rest of the paper.

The basic parameters describe the average treatment effects across all studies of all designs, and so the entire evidence base, of treatments relative to the reference treatment *A*. We take the difference between two basic parameters to make inferences about other average treatments. For example the estimated average effect of treatment *E* relative to treatment *B* is given by ${\hat{\delta }}^{\mathrm{AE}}-{\hat{\delta }}^{\mathrm{AB}}$. Upon approximating the two unknown variances with their estimates, model (2) assumes that the outcome data are multivariate normal with a known covariance matrix. Hence, $\hat{\delta }$ is also treated as multivariate normal with known covariance matrix, and making inferences about any linear combination of the basic parameters is straightforward.

## 3 PREVIOUSLY PROPOSED ESTIMATION METHODS

A variety of estimation methods for our model have previously been proposed. We will briefly describe these alternative methods before describing the new Paule and Mandel estimation method. The details given here for the previously proposed estimation methods are necessarily concise, and so we refer the reader to the original methodological papers referred to below for full details.

### 3.1 The “DerSimonian and Laird” estimation method

The DerSimonian and Laird^4^ estimator for standard univariate random‐effects meta‐analysis is based on the *Q* statistic. To extend this estimation method to the network meta‐analysis setting, Jackson et al^14^ define the scalar *Q* statistic for the entire network meta‐analysis as

(4) $$Q^{\;\mathrm{net}\;}={\left(Y-\hat{Y}\right)}^{T}S^{-1}\left(Y-\hat{Y}\right),$$

where $\hat{Y}=X{\left(X^{T}S^{-1}X\right)}^{-1}X^{T}S^{-1}Y$ is the fitted outcome vector under model (2) with $\tau_{\beta }^{2}=\tau_{\omega }^{2}=0$, that is, the common‐effect and consistent model. This is simply the usual *Q* scalar heterogeneity statistic that is also used in multivariate meta‐regression.^26^, ^27^ However the multivariate meta‐regression model is not the same as the network meta‐analysis model used here. Hence, the distribution of *Q* ^*net*^ is different under these two models because of the different types of assumptions made in these contexts.

The decomposition of this *Q* statistic that was proposed by Krahn et al,^28^ and used for estimation purposes by Jackson et al,^14^ is

(5) $$Q^{\;\mathrm{net}\;}=\sum_{d=1}^{D}‍Q_{d}^{\;\mathrm{het}\;}+Q^{\;\mathrm{inc}\;},$$

where *D* is the number of different designs present. Here, $Q_{d}^{\;\mathrm{het}\;}$ is the *Q* statistic defined as in Equation (4) but we use only data from design *d*. That is,

(6) $$Q_{d}^{\;\mathrm{het}\;}={\left(Y_{d}-{\hat{Y}}_{d}\right)}^{T}S_{d}^{-1}\left(Y_{d}-{\hat{Y}}_{d}\right),$$

where ${\hat{Y}}_{d}$ is the fitted outcome vector for design *d*, under the common‐effect and consistent model and using data only from this design. The final term in Equation (5), *Q*^*inc*^ , is obtained by subtraction.

Jackson et al^14^ show that the expectation of *Q* ^*net*^ is a linear equation in $\tau_{\beta }^{2}$ and $\tau_{\omega }^{2}$, and the expectation of $\sum_{d=1}^{D}‍Q_{d}^{\;\mathrm{het}\;}$ is linear in $\tau_{\beta }^{2}$. Under the consistency assumption they suggest matching *Q* ^*net*^ to its expectation to estimate $\tau_{\beta }^{2}$. Under the full model they suggest matching $\sum_{d=1}^{D}‍Q_{d}^{\;\mathrm{het}\;}$ to its expectation to estimate $\tau_{\beta }^{2}$. The latter estimate can then be substituted into the expectation of *Q* ^*net*^ which similarly gives rise to the estimate of $\tau_{\omega }^{2}$ by matching moments. Any negative estimated variance components are truncated to 0.

### 3.2 Likelihood‐based estimation methods

Law et al^15^ explain how the *rma.mv* command in the *R* package *metafor* 29 can be used to perform likelihood‐based inference under the full model and the consistency model ( $\tau_{\omega }^{2}=0$). By using normal approximations, the likelihood function can be evaluated and maximised numerically to simultaneously estimate all parameters.

However, the sample size in many meta‐analyses is small.^30^ Maximum likelihood‐based estimates of unknown variance components, such as the two included in our model, are generally biased downwards in small datasets. REML estimation helps to overcome this difficulty and so is generally recommended. The restricted likelihood function eliminates the location parameters (here, these are the basic parameters) and this function can also be maximised numerically to estimate the unknown variance components.

Bayesian estimation has also been proposed. Bayesian analyses are also based on the likelihood but make more assumptions than classical analyses, where these additional assumptions are made via the prior distributions. Analytical solutions are difficult to obtain, so computationally more expensive methods have been proposed. Jackson et al^13^ provide WinBUGS code for fitting the model. Law et al^15^ also use WinBUGS and develop importance sampling algorithms that assume lognormal priors for the unknown variance components. An advantage of the Bayesian approach is that the uncertainty in the two unknown variance components is fully taken into account when making inferences about the treatment effects. In this paper, however, we will focus on classical methods.

## 4 THE PROPOSED PAULE AND MANDEL ESTIMATION METHOD FOR NETWORK META‐ANALYSIS

To motivate our Paule and Mandel estimation method for network meta‐analysis, we begin by describing this established and recommended method for univariate meta‐analysis.

### 4.1 Univariate random‐effects meta‐analysis

In a univariate meta‐analysis, each study provides a single estimate *Y*_*i*_ of the same outcome or treatment comparison. The random‐effects model for meta‐analysis is *Y*_*i*_ = *δ* + *B*_*i*_ + *ε*_*i*_, where δ is the average effect, *B*_*i*_ ~ *N*(0, *τ*^2^) describes the between‐study heterogeneity and $\varepsilon_{i}\sim N\left(0,\sigma_{i}^{2}\right)$. This model is a much simpler version of model (1).

The quantity *Q* used in the Paule and Mandel estimation method is a function of *τ*^2^, and we will emphasise this dependence by writing this *Q* as

(7) $$Q\left(\tau^{2}\right)=\sum_{i=1}^{n}‍w_{i}\left(\tau^{2}\right){\left(Y_{i}-\hat{\delta }\left(\tau^{2}\right)\right)}^{2}\sim \chi_{n-1}^{2},$$

where $w_{i}\left(\tau^{2}\right)=1/\left(\sigma_{i}^{2}+\tau^{2}\right)$, $\hat{\delta }\left(\tau^{2}\right)=\sum_{i=1}^{n}‍w_{i}\left(\tau^{2}\right)Y_{i}/\sum_{i=1}^{n}‍w_{i}\left(\tau^{2}\right)$ and *n* is the number of studies. *Q*(*τ*^2^) is not a statistic because it is a function of the unknown parameter *τ*^2^. However, it is a pivot, and so we can use the distributional result in Equation (7) to make inferences.

To estimate *τ*^2^, we take the expectation of Equation (7) and replace *τ*^2^ with its estimate to obtain ${\hat{\tau }}^{2}$ as the solution to

(8) $$Q\left({\hat{\tau }}^{2}\right)=n-1$$

Equation (8) is non‐linear and must be solved numerically. This is also the case for the Paule and Mandel estimators for network meta‐analysis that follow, but this presents very little difficulty in practice. This is because *Q*(*τ*^2^) is a continuous and strictly decreasing function in *τ*^2^ for the observed data,^13^, ^31^, ^32^ and $\lim_{\tau^{2}\to \infty }Q\left(\tau^{2}\right)=0^{+}$. Hence, if *Q*(0) > *n* − 1, then a unique estimate arises from solving Equation (8). Otherwise, ${\hat{\tau }}^{2}$ is truncated to 0, on the grounds that the variation in the data is even less than what would be expected if *τ*^2^ = 0. Paule and Mandel estimators have also been developed for meta‐regression models.^33^

Let us compare the quadratic form used by the Paule and Mandel method in Equation (7) to the *Q* statistic used in the more familiar DerSimonian and Laird estimation procedure for univariate meta‐analysis, which is given by

(9) $$Q=Q\left(0\right)=\sum_{i=1}^{n}‍\sigma_{i}^{-2}{\left(Y_{i}-\hat{\delta }\right)}^{2}$$

where $\hat{\delta }=\sum_{i=1}^{n}‍\sigma_{i}^{-2}Y_{i}/\sum_{i=1}^{n}‍\sigma_{i}^{-2}$. The salient observation is that the DerSimonian and Laird method is based on a special case of the *Q*(*τ*^2^) quantity used in the Paule and Mandel estimation method, where the unknown *τ*^2^ is set to 0 in the DerSimonian and Laird weights in Equation (9). The DerSimonian and Laird quadratic form therefore weights by the within‐study precisions and the Paule‐Mandel method instead weights by the total precisions, that is the reciprocals of the sums of the within and the between‐study variances.

DerSimonian and Laird type *Q* statistics for network meta‐analysis, which can be used to estimate the unknown variance components, were described above in Section 3.1. We will similarly define Paule and Mandel versions of these *Q* statistics below. As in the univariate case, the Paule and Mandel weights will be the total precision, rather than the within‐study precisions **S**^−1^ and $S_{d}^{-1}$, in Equations (4) and (6), respectively.

### 4.2 Paule‐Mandel estimators for network meta‐analysis

To extend the univariate Paule and Mandel estimators to the network meta‐analysis setting, we must define some more general versions of Equations (7) and (8) that are suitable for network meta‐analysis. These will be inspired by the DerSimonian and Laird *Q* statistics (Equations (4) and (6)) and the connection between the univariate DerSimonian and Laird and Paule and Mandel quadratic forms described above. We begin by explaining how to estimate $\tau_{\beta }^{2}$ under the consistency assumption. This is because estimation is much simpler under this assumption. Hence, we describe this estimation to make this option available but, with the exception of the further investigation of the discrepancies between our results for one of our examples below, we do not apply it in this paper.

#### 4.2.1 The first Paule‐Mandel pivot for network meta‐analysis: estimating $\tau_{\beta }^{2}$ under the consistency assumption

Equation (4) is the DerSimonian and Laird *Q* statistic for the entire network, where the weights are the within‐study precision **S**^−1^. Jackson et al^14^ suggest using Equation (4) to estimate $\tau_{\beta }^{2}$ under the consistency assumption. Under the consistency assumption, the total precision is ${\left(\tau_{\beta }^{2}M_{1}+S\right)}^{-1}$. This observation and the relationship between the DerSimonian and Laird and Paule and Mandel quadratic forms described at the end of Section 4.1 suggests using the pivot

(10) $$Q^{\;\mathrm{net}\;}\left(\tau_{\beta }^{2}\right)={\left(Y-\hat{Y}\left(\tau_{\beta }^{2}\right)\right)}^{T}{\left(\tau_{\beta }^{2}M_{1}+S\right)}^{-1}\left(Y-\hat{Y}\left(\tau_{\beta }^{2}\right)\right)\sim \chi_{n-c}^{2}$$

where $\hat{Y}\left(\tau_{\beta }^{2}\right)=X{\left(X^{T}{\left(\tau_{\beta }^{2}M_{1}+S\right)}^{-1}X\right)}^{-1}X^{T}{\left(\tau_{\beta }^{2}M_{1}+S\right)}^{-1}Y$, *n* is now the number of estimates (the length of **Y**, which is equal to the number of studies if and only if all studies include 2 treatment groups) and *c* continues to be the number of basic parameters. The $\chi_{n-c}^{2}$ distributional statement in Equation (10) is correct under model (2) and the consistency assumption, ie, $\tau_{\omega }^{2}=0$ so that all **ω**_d_ = **0**. The quadratic form in Equation (10) is very similar to Equation (4) but where the total, rather than just the within‐study, precision under the consistency model is used as the weight. This is the same as the relationship the between the quadratics used in the univariate estimation methods described in Section 4.1. Briefly, the χ^2^ distributional statement in Equation (10), and those that follow below are because if the outcome data **Y** follow a multivariate normal distribution, whose mean depends linearly on *c* unknown location parameters and the corresponding covariance matrix **V** is given, then ${\left(Y-\hat{Y}\right)}^{T}V^{-1}\left(Y-\hat{Y}\right)\sim \chi_{n-c}^{2}$.

Upon taking the expectation of Equation (10) and replacing $\tau_{\beta }^{2}$ with its estimate under the consistency assumption, in the same way as in Equations (7) and (8) when deriving the Paule and Mandel estimator for univariate meta‐analysis, we obtain the estimating equation

(11) $$Q^{\;\mathrm{net}\;}\left({\hat{\tau }}_{\beta ,\mathrm{con}\;}^{2}\right)=n-c$$

This estimating equation provides an analogous Paule and Mandel type estimate to the DerSimonian and Laird estimate of $\tau_{\beta }^{2}$ under the consistency assumption. The expectation of quadratic forms of this type does not require normality assumptions (Searle ^p.55,^ 34). Hence, the Paule and Mandel estimators are semiparametric in the sense that they provide estimates of the unknown variance components without requiring normality.

We will establish below that if *Q* ^*net*^ (0) > *n* − *c* then the solution to Equation (11) is unique, and otherwise, we truncate ${\hat{\tau }}_{\beta ,\mathrm{con}\;}^{2}=0$ for the same reasons as in the univariate case.

#### 4.2.2 The second Paule‐Mandel pivot for network meta‐analysis: estimating $\tau_{\beta }^{2}$

The estimate of $\tau_{\beta }^{2}$ in Section 4.2.1 is valid only under the consistency assumption. Our intention is to relax this assumption, and so we require an alternative estimate that is valid under the full model. Using similar arguments as in the previous section, Equation (6) suggests that the use of the design‐specific pivot

(12) $$Q_{d}^{\;\mathrm{het}\;}\left(\tau_{\beta }^{2}\right)={\left(Y_{d}-{\hat{Y}}_{d}\left(\tau_{\beta }^{2}\right)\right)}^{T}{\left(\tau_{\beta }^{2}M_{d}+S_{d}\right)}^{-1}\left(Y_{d}-{\hat{Y}}_{d}\left(\tau_{\beta }^{2}\right)\right)\sim \chi_{\left(n_{d}-1\right)c_{d}}^{2}$$

where ${\hat{Y}}_{d}\left(\tau_{\beta }^{2}\right)=X_{d}{\left(X_{d}^{T}{\left(\tau_{\beta }^{2}M_{d}+S_{d}\right)}^{-1}X_{d}\right)}^{-1}X_{d}^{T}{\left(\tau_{\beta }^{2}M_{d}+S_{d}\right)}^{-1}Y_{d}$. The $\chi_{\left(n_{d}-1\right)c_{d}}^{2}$ distribution in Equation (12) follows from submodel (3) that is used to describe data from a single design; there are *n*_*d*_*c*_*d*_ treatment effects from studies of design *d*, and *c*_*d*_ location parameters are estimated (the entries of *β*_*d*_), so the degrees of freedom are *n*_*d*_*c*_*d*_ − *c*_*d*_ = (*n*_*d*_ − 1)*c*_*d*_.

Taking the sum of the $Q_{d}^{\;\mathrm{het}\;}\left(\tau_{\beta }^{2}\right)$ and then the expectation results in the estimating equation,

(13) $$\sum_{d=1}^{D}‍Q_{d}^{\;\mathrm{het}\;}\left({\hat{\tau }}_{\beta }^{2}\right)=\sum_{d=1}^{D}‍\left(n_{d}-1\right)c_{d}$$

This estimating equation provides an analogous Paule and Mandel type estimate to the DerSimonian and Laird estimate of $\tau_{\beta }^{2}$ under the full model. We will establish below that if $\sum_{d=1}^{D}‍Q_{d}^{\;\mathrm{het}\;}\left(0\right)>\left(n_{d}-1\right)c_{d}$ then the solution to Equation (13) is unique, and otherwise, we truncate ${\hat{\tau }}_{\beta }^{2}=0$.

#### 4.2.3 The third Paule‐Mandel pivot for network meta‐analysis: estimating $\tau_{\omega }^{2}$

Jackson et al^14^ also use *Q*^*net*^ to provide a further estimating equation in both $\tau_{\beta }^{2}$ and $\tau_{\omega }^{2}$ and so estimate $\tau_{\omega }^{2}$ after estimating $\tau_{\beta }^{2}$ (although this process is equivalent to solving the pair of simultaneous linear equations, as explained by Jackson et al^14^) The total precision under the full model is ${\left(\tau_{\beta }^{2}M_{1}+\tau_{\omega }^{2}M_{2}+S\right)}^{-1}$ so that similar arguments as above suggest using the pivot

(14) $$Q^{\;\mathrm{net}\;}\left(\tau_{\beta }^{2},\tau_{\omega }^{2}\right)={\left(Y-\hat{Y}\left(\tau_{\beta }^{2},\tau_{\omega }^{2}\right)\right)}^{T}{\left(\tau_{\beta }^{2}M_{1}+\tau_{\omega }^{2}M_{2}+S\right)}^{-1}\left(Y-\hat{Y}\left(\tau_{\beta }^{2},\tau_{\omega }^{2}\right)\right)\sim \chi_{n-c}^{2},$$

where $\hat{Y}\left(\tau_{\beta }^{2},\tau_{\omega }^{2}\right)=X{\left(X^{T}{\left(\tau_{\beta }^{2}M_{1}+\tau_{\omega }^{2}M_{2}+S\right)}^{-1}X\right)}^{-1}X^{T}{\left(\tau_{\beta }^{2}M_{1}+\tau_{\omega }^{2}M_{2}+S\right)}^{-1}Y$. Taking the expectation of Equation (14) results in the estimating equation

(15) $$Q^{\;\mathrm{net}\;}\left({\hat{\tau }}_{\beta }^{2},{\hat{\tau }}_{\omega }^{2}\right)=n-c$$

As in the previously proposed DerSimonian and Laird estimating procedure, we suggest solving Equations (13) and (15) simultaneously to estimate $\tau_{\beta }^{2}$ and $\tau_{\omega }^{2}$ under the full model. These equations can easily be solved because estimating Equation (13) depends on only one estimate and immediately leads to ${\hat{\tau }}_{\beta }^{2}$, which can be substituted into Equation (15). Hence, when estimating the inconsistency variance $\tau_{\omega }^{2}$, our proposal is effectively to use the pivot in Equation (14) with $\tau_{\beta }^{2}={\hat{\tau }}_{\beta }^{2}$. Therefore, $\tau_{\beta }^{2}$ is held fixed at its estimate from Equation (13) when estimating $\tau_{\omega }^{2}$ using Equation (15). Equation (15) has multiple solutions but there can only be one solution that also satisfies Equation (13), and we take the solutions of these two simultaneous non‐linear equations as our estimates.

We establish below that if $Q^{\;\mathrm{net}\;}\left({\hat{\tau }}_{\beta }^{2},0\right)>n-c$ then the resulting positive estimate ${\hat{\tau }}_{\omega }^{2}$ is unique. Otherwise, we take ${\hat{\tau }}_{\omega }^{2}=0$, again following similar arguments as before, so that unique estimates of both unknown variance components are obtained.

### 4.3 Ensuring unique solutions to the estimating equations

To ensure that the estimates from Equations (11), (13), and (15) are unique, we must establish that the 3 pivots (Equations (10), (12), and (14)) are strictly decreasing in the variance parameter to be estimated from them. This is because if these pivots are not strictly decreasing in this way then the estimating equations (Equations (11), (13), and (15)) could instead have multiple solutions. In fact, we require only that the sum of the pivots $Q_{d}^{\;\mathrm{het}\;}\left(\tau_{\beta }^{2}\right)$ in Equation (12) are strictly decreasing in $\tau_{\beta }^{2}$ to ensure a unique solution to Equation (13), but we will establish the stronger condition that all $Q_{d}^{\;\mathrm{het}\;}\left(\tau_{\beta }^{2}\right)$ are strictly decreasing in $\tau_{\beta }^{2}$.

This type of strictly decreasing property has already been shown for the pivots used in meta‐analysis^31^, ^32^ and meta‐regression,^33^ but in the presence of multi‐arm studies, matters are more complicated because then the estimates are no longer independent. To ensure that estimates can always be obtained, we also require that the pivots are continuous and differentiable in the variance parameter to be estimated and also that the pivots tend towards zero as this variance becomes large. These extra conditions, in conjunction with the condition that the pivots are strictly decreasing, ensure that unique solutions to the estimating equations can always be found (although sometimes we will need to truncate the estimated variance components to zero). This is because then if Equations (11), (13), or (15) are more than their associated degrees of freedom when taking the unknown variance to be zero, we can simply increase the unknown variance until the corresponding estimating equation is satisfied; if Equation (11), (13), or (15) is instead less than or equal to their degrees of freedom when taking the unknown variance to be 0, then we truncate the estimate to 0. Hence, with all these conditions established, even very simple numerical methods can be safely used to solve, the estimating equations and there are no possible convergence problems.

It is easy to establish that both of these additional conditions are satisfied. Firstly, all values that contribute to the computation of the *Q* pivots are continuous and differentiable in the variance parameter to be estimated; hence so are the pivots. Secondly, as an unknown variance becomes large, the weights in the regressions become dominated by the **M** matrix associated with this variance. Hence, the fitted values, and so the residuals, tend towards the corresponding values from the regression where the weights are the inverse of **M**. Furthermore, as the unknown variance becomes large, the entries of precision matrices (the inverses of the total variances that appear in Equations (10), (12), and (14)) tend towards 0. With the residuals stable and the entries of the precision matrices tending towards 0, all three *Q* pivots used for estimation tend towards 0 as the variance to be estimated using them becomes large.

Proving that all three types of *Q* pivot are strictly decreasing in the unknown variance to be estimated is more difficult. However, we provide this proof in the Appendix A. The Paule and Mandel estimates are therefore uniquely defined by the estimating equations.

## 5 APPLICATIONS

We now apply all three classical estimation methods to a variety of real examples. In particular, it will be interesting to compare the results using the new Paule and Mandel estimators to those using the previously proposed extension of DerSimonian and Laird's method^14^ and REML.^15^

### 5.1 Application to three previous examples

We begin by applying all three classical estimation methods to some examples that have been used previously. The first and second examples were used by Law et al,^15^ and the second and third examples were used by Jackson et al.^14^ Briefly, the first example concerns treatments for prostate cancer. Here, there are 8 treatments, where the outcome is all‐cause mortality, where a negative relative effect (log odds ratio) indicates treatment benefit. The second example concerns treatments for chronically discharging ears. Here, there are 4 treatments for treating discharge, where a negative relative effect (log odds ratio) indicates treatment benefit. In the first and second examples, the outcome is binary and we use conventional normal approximations to the log odds ratios used in analysis. The third example concerns treatments for osteoarthritis of the knee (OAK). Here, there are 22 treatments for pain relief, where a negative relative effect (standardised mean difference) indicates treatment benefit. See Jackson et al^14^ and Law et al^15^ for more details of these datasets and their supplementary materials that provide the data.

The estimated unknown variance components are shown in Table 1. The approximate 95% confidence intervals corresponding to the REML estimates were obtained using the profile likelihood and the code provided by Law et al.^15^ This method for calculating confidence intervals when using REML is an advantage of using a likelihood based approach. We return to the issue of how these confidence intervals might be obtained using the other two estimation methods in the discussion. Given the uncertainty in the point estimates of the unknown variance components, the results in Table 1 are in broad agreement but there are also notable differences between the estimates from the three methods. This suggests that all three estimation methods perform satisfactorily for these examples. This is also the case for the basic parameters (results shown in Supplementary data files S1 and S2).

**Table 1:** Estimated unknown variance components for 3 previously used examples, using 3 different estimation methods

Dataset	PM		DL		REML			
	${\hat{\tau }}_{\beta }^{2}$	${\hat{\tau }}_{\omega }^{2}$	${\hat{\tau }}_{\beta }^{2}$	${\hat{\tau }}_{\omega }^{2}$	${\hat{\tau }}_{\beta }^{2}$	${\hat{\tau }}_{\omega }^{2}$	95% CI: $\tau_{\beta }^{2}$	95% CI: $\tau_{\omega }^{2}$
PC	0	0	0	0	0	0	(0, 0.07)	(0, 0.62)
CDE	0.36	0.52	0.25	0.30	0.10	0.54	(0, 1.67)	(0, 3.96)
OAK	0.35	0	0.18	0	0.18	0	(0.09, 0.31)	(0, 0.12)

Point estimates of $\tau_{\beta }^{2}$ and $\tau_{\omega }^{2}$ are given for all 3 methods. Approximate 95% confidence intervals (CIs) using REML are also given and are obtained from the profile likelihood.

Abbreviations: PM, the proposed Paule and Mandel estimation method; DL, a generalisation of DerSimonian and Laird's univariate method; REML, restricted maximum likelihood.

The agreement between the results is particularly strong for the first example where all methods result in ${\hat{\tau }}_{\beta }^{2}={\hat{\tau }}_{\omega }^{2}=0$, and so the same inference, as appropriate for this highly homogenous example.^15^ Given the extent of the parameter uncertainty, perhaps the most notable disagreement between the results is for the third example. Although all estimation methods provide ${\hat{\tau }}_{\omega }^{2}=0$, which indicates some good agreement, the proposed Paule and Mandel method provides a much larger estimate of ${\hat{\tau }}_{\beta }^{2}=0.35$. This estimate lies just above the REML confidence interval of (0.09, 0.31). The Bayesian results of Jackson et al^13^ are in better agreement with the DerSimonian and Laird method and REML.

However OAK is a challenging example involving many treatments and relatively little replication within designs (as Jackson et al^14^ explain that there are 87 studies and 38 designs), which makes the identification of $\tau_{\beta }^{2}$ in the full model challenging. Under the consistency assumption, which is supported by the three ${\hat{\tau }}_{\omega }^{2}=0$, the estimates of $\tau_{\beta }^{2}$ are Paule and Mandel: 0.25; DerSimonian and Laird: 0.15; REML: 0.18, 95% CI, (0.10‐0.31). The REML point estimate of $\tau_{\beta }^{2}$ is the same under the consistency and the full model because REML provides ${\hat{\tau }}_{\omega }^{2}=0$. However, the two moments based estimates differ under these two models, despite all ${\hat{\tau }}_{\omega }^{2}=0$, because different estimating equations for $\tau_{\beta }^{2}$ are used under the consistency and full models when using these two methods. When using the proposed Paule and Mandel method, $\tau_{\beta }^{2}$ is estimated under these two models using Equations (11) and (13), respectively. This is discussed for the DerSimonian and Laird estimator in Jackson et al.^14^

Upon using the information contained in the consistency assumption so that replication within designs is not needed when estimating $\tau_{\beta }^{2}$, the Paule and Mandel estimator is in much better agreement with the other methods. The discrepancy between the Paule and Mandel estimate of $\tau_{\beta }^{2}$ for the OAK data appears to be largely because of the difficulty in estimating $\tau_{\beta }^{2}$ in the full model, which is due to the relative lack of replication within designs. We conclude that the overall picture is that the results from the three estimation methods are reasonably compatible, despite the notable differences that have been observed. Our results indicate that all estimation methods have performed satisfactorily for these three examples, but it is also evident that alternative estimation methods can result in important differences in practice. Applied meta‐analysts may therefore wish to explore the use of all three estimation methods, to determine how robust their inferences are to their choice of estimation procedure.

### 5.2 Application to a challenging new example

**Figure 1:**
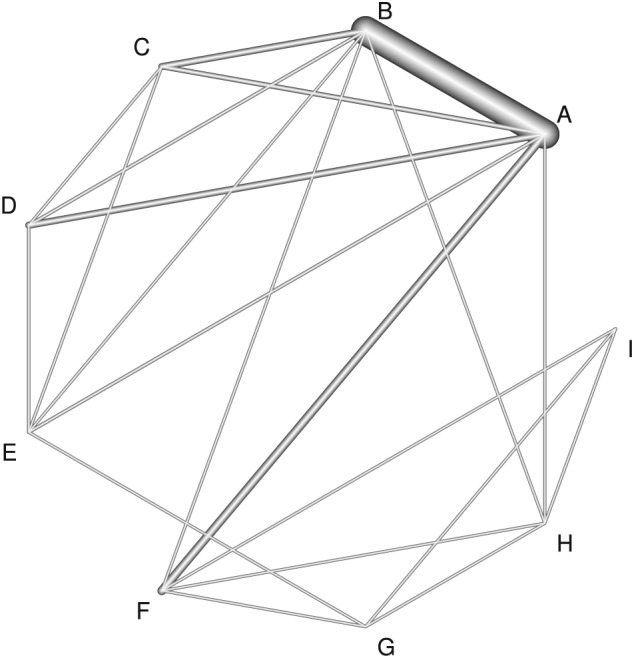
Network diagram for the Alzheimer's dementia dataset. The thickness of the lines is proportional to the number of direct comparisons between each pair of treatments. (A) Placebo, (B) donepezil, (C) galantamine, (D) rivastigmine oral, (E) rivastigmine patch, (F) memantine, (G) rivastigmine patch and memantine, (H) donepezil and memantine, and (I) galantamine and memantine

We now apply our methods to a challenging new example from Tricco et al.^35^ This concerns the comparative effectiveness of cognitive enhancers for treating Alzheimer's dementia, and we focus on data from one outcome of several considered by Tricco et al, the mini‐mental state examination. Tricco et al^35^ impute standard deviations for studies that do not report these; however, here, we restrict analysis to studies where this imputation is not necessary, to provide a dataset where inconsistency is more evident and so better illustrate our methods. This results in 41 studies, 37 of which compare 2 treatments, 3 of which compare 3 treatments and a single 4‐arm study. There are 9 treatments in the network: (A) placebo, (B) donepezil, (C) galantamine, (D) rivastigmine oral, (E) rivastigmine patch, (F) memantine, (G) rivastigmine patch and memantine, (H) donepezil and memantine, and (I) galantamine and memantine. The mean difference was used as the outcome, where a positive difference indicates treatment benefit. The within‐study covariance structure was calculated without the unnecessary assumption that the standard deviation is the same in arms from the same study. For example, in a 2‐arm study with reported standard deviations of *s*_1_ and *s*_2_ and sample sizes of *N*_1_ and *N*_2_, the within‐study variance was computed as $s_{1}^{2}/N1+s_{2}^{2}/N_{2}$. A network diagram is shown in Figure 1, where the thickness of the lines are proportional to the number of direct comparisons between each treatment pair. The absence of a line indicates that there is no direct comparison. This Figure was produced using the *netmeta* package^36^ in R.

As we will see below, these data provide large estimates of $\tau_{\beta }^{2}$ and $\tau_{\omega }^{2}$. However, they are also challenging because the study sizes differ greatly. There are some very small studies present (for example, the 3 smallest studies' total numbers of patients are just 14, 17, and 20) and some much larger studies (the largest study has 2 arms and 1765 patients). Hence, these data are very heterogeneous both in the study sizes and the mini‐mental state examination study results. There is also relatively little data (46 estimates) to estimate 10 parameters (8 basic parameters and 2 unknown variances). These data therefore present a very considerable challenge to the 3 estimation methods.

Using the proposed Paule and Mandel estimation method we obtain ${\hat{\tau }}_{\beta }^{2}=0.51$ and ${\hat{\tau }}_{\omega }^{2}=0.91$. These estimates indicate that there is substantial between‐study heterogeneity and inconsistency. These findings are corroborated by the REML estimates of ${\hat{\tau }}_{\beta }^{2}=0.68$ and ${\hat{\tau }}_{\omega }^{2}=0.54$, with 95% confidence intervals of (0.31, 1.55) and (0, 2.94), respectively. The wide REML confidence interval for $\tau_{\omega }^{2}$ indicates that the inconsistency variance is difficult to identify and because this interval contains 0 and that there is insufficient evidence to reject the null hypothesis that the consistency assumption is true.

The DerSimonian and Laird method provides somewhat different estimates of ${\hat{\tau }}_{\beta }^{2}=1.20$ and ${\hat{\tau }}_{\omega }^{2}=0$, so that the fitted model collapses to the consistency model. However, these estimates lie within the REML confidence intervals and so are also reasonably consistent with the likelihood based results. This difference in the estimated covariance structure results in slightly different point estimates of effect and markedly smaller standard errors, as shown in Table 2. In this table, we show the estimated effects for all pairwise comparisons. To obtain the standard error for comparisons that do not involve treatment *A*, we require the covariance matrix of the basic parameters. This covariance matrix is provided by all sets of computing codes, and as the matrix “vb” in the object created by the *rma.mv* command from the *metafor* package^29^ when using REML. See Law et al^15^ for examples of computing codes that use the *rma.mv* command.

**Table 2:** Estimated average treatment effects for the Alzheimer's dementia data using 3 different estimation methods

Comparison	Parameters	PM	DL	REML
AB	*δ*^*AB*^	0.60 (0.59)	0.71 (0.27)	0.60 (0.51)
AC	*δ*^*AC*^	0.18 (0.69)	0.31 (0.49)	0.21 (0.61)
AD	*δ*^*AD*^	0.09 (0.60)	0.19 (0.44)	0.11 (0.53)
AE	*δ*^*AE*^	1.72 (0.64)	1.75 (0.57)	1.71 (0.59)
AF	*δ*^*AF*^	0.93 (0.79)	0.79 (0.52)	0.89 (0.70)
AG	*δ*^*AG*^	1.58 (1.04)	1.56 (0.93)	1.56 (0.97)
AH	*δ*^*AH*^	2.23 (0.88)	2.22 (0.78)	2.20 (0.82)
AI	*δ*^*AI*^	2.16 (1.31)	2.10 (1.18)	2.13 (1.22)
BC	*δ*^*AC*^ − *δ*^*AB*^	−0.42 (0.66)	−0.40 (0.49)	−0.39 (0.59)
BD	*δ*^*AD*^ − *δ*^*AB*^	−0.51 (0.66)	−0.52 (0.48)	−0.49 (0.60)
BE	*δ*^*AE*^ − *δ*^*AB*^	1.13 (0.75)	1.04 (0.60)	1.12 (0.69)
BF	*δ*^*AF*^ − *δ*^*AB*^	0.34 (0.86)	0.08 (0.57)	0.29 (0.77)
BG	*δ*^*AG*^ − *δ*^*AB*^	0.99 (1.08)	0.85 (0.95)	0.96 (1.01)
BH	*δ*^*AH*^ − *δ*^*AB*^	1.63 (0.88)	1.51 (0.78)	1.60 (0.82)
BI	*δ*^*AI*^ − *δ*^*AB*^	1.56 (1.33)	1.39 (1.19)	1.53 (1.25)
CD	*δ*^*AD*^ − *δ*^*AC*^	−0.09 (0.76)	−0.12 (0.61)	−0.10 (0.69)
CE	*δ*^*AE*^ − *δ*^*AC*^	1.55 (0.82)	1.45 (0.70)	1.51 (0.76)
CF	*δ*^*AF*^ − *δ*^*AC*^	0.76 (0.98)	0.48 (0.71)	0.68 (0.88)
CG	*δ*^*AG*^ − *δ*^*AC*^	1.41 (1.16)	1.25 (1.03)	1.35 (1.08)
CH	*δ*^*AH*^ − *δ*^*AC*^	2.05 (1.02)	1.91 (0.90)	1.99 (0.95)
CI	*δ*^*AI*^ − *δ*^*AC*^	1.98 (1.41)	1.79 (1.26)	1.92 (1.32)
DE	*δ*^*AE*^ − *δ*^*AD*^	1.64 (0.68)	1.56 (0.61)	1.61 (0.63)
DF	*δ*^*AF*^ − *δ*^*AD*^	0.85 (0.93)	0.60 (0.68)	0.78 (0.84)
DG	*δ*^*AG*^ − *δ*^*AD*^	1.50 (1.10)	1.37 (0.99)	1.45 (1.03)
DH	*δ*^*AH*^ − *δ*^*AD*^	2.14 (0.98)	2.03 (0.87)	2.09 (0.92)
DI	*δ*^*AI*^ − *δ*^*AD*^	2.07 (1.38)	1.91 (1.24)	2.02 (1.29)
EF	*δ*^*AF*^ − *δ*^*AE*^	−0.79 (0.94)	−0.96 (0.74)	−0.83 (0.86)
EG	*δ*^*AG*^ − *δ*^*AE*^	−0.14 (1.00)	−0.20 (0.92)	−0.16 (0.94)
EH	*δ*^*AH*^ − *δ*^*AE*^	0.51 (0.99)	0.46 (0.91)	0.48 (0.93)
EI	*δ*^*AI*^ − *δ*^*AE*^	0.43 (1.36)	0.35 (1.25)	0.41 (1.27)
FG	*δ*^*AG*^ − *δ*^*AF*^	0.65 (1.09)	0.77 (0.97)	0.67 (1.02)
FH	*δ*^*AH*^ − *δ*^*AF*^	1.30 (0.97)	1.43 (0.84)	1.31 (0.91)
FI	*δ*^*AI*^ − *δ*^*AF*^	1.22 (1.26)	1.31 (1.17)	1.24 (1.19)
GH	*δ*^*AH*^ − *δ*^*AG*^	0.65 (1.10)	0.66 (1.03)	0.64 (1.04)
GI	*δ*^*AI*^ − *δ*^*AG*^	0.57 (1.31)	0.54 (1.23)	0.57 (1.24)
HI	*δ*^*AI*^ − *δ*^*AH*^	−0.07 (1.26)	−0.12 (1.17)	−0.07 (1.19)

Standard errors are given in parentheses. (A) placebo, (B) donepezil, (C) galantamine, (D) rivastigmine oral, (E) rivastigmine patch, (F) memantine, (G) rivastigmine patch and memantine, (H) donepezil and memantine, and (I) galantamine and memantine.

Abbreviations: PM, the proposed Paule and Mandel estimation method; DL, A generalisation of DerSimonian and Laird's univariate method; REML, restricted maximum likelihood.

There is robust evidence in this network that treatments E and H are better than placebo. We will use this final dataset, and the corresponding REML estimates, to motivate a simulation study in Section 6, to examine the 3 methods in more detail and also to determine which method performs best in these challenging circumstances.

## 6 SIMULATION STUDY

It is interesting that the DerSimonian and Laird method attributed all the excess variation in the previous example to between‐study heterogeneity. The other two methods instead attributed this to a mixture of between‐study heterogeneity and inconsistency. To better understand why this might be the case and also to compare the different methods in a challenging and real setting, a simulation study based on this example was performed.

### 6.1 Simulation study design

As explained above, the study sizes differ considerably in this example, and so it is difficult to determine a typical study size. This difficulty is reflected in the notably different measures of a typical study size for this example: the mean univariate within‐study variance is 0.53, the median is 0.29 and the representative within‐study variance of Higgins and Thompson^37^ is just 0.02. We decided to base our simulation study on the REML point estimates, whilst also allowing for a variety of variance structures, and so examined 5 different values of $\tau_{\beta }^{2}=0,0.25,0.5,0.75,1$. This explores a wide range of between‐study heterogeneities in relation to the study sizes and includes the REML estimate ${\hat{\tau }}_{\beta }^{2}=0.68$ within the range considered.

The REML estimate of ${\hat{\tau }}_{\omega }^{2}=0.54$ is large, and so we examined 3 different values of $\tau_{\omega }^{2}=0,0.25,0.5$, to provide 15 simulation settings. As explained by Jackson et al,^14^ very large inconsistencies should discourage the use of network meta‐analysis per se, and so we did not explore the use of larger inconsistency variances such as 0.75 and 1, as used for $\tau_{\beta }^{2}$, or the large point estimate of $\tau_{\omega }^{2}$ that was obtained using the Paule and Mandel method. Our 3 values of $\tau_{\omega }^{2}$ cover a wide range of possibilities where the use of network meta‐analysis is likely to be encouraged. In particular, the combination of $\tau_{\beta }^{2}=0.75$ and $\tau_{\omega }^{2}=0.5$ reflects the REML estimates for the real example, and we also explore a range of other possibilities that are compatible with the real data and also represent situations where the data are not so inconsistent as to entirely discourage network meta‐analysis.

We simulated data directly from model (2) assuming that all basic parameters are 0; however, this choice of ***δ*** is immaterial because the estimation of the unknown variance components is location invariant and the estimates of the basic parameters are directly shifted by using other values. To simulate data in an especially transparent way and also to simulate data when the assumed model is true, we take the within‐study covariance matrix **S** be its numerical value from the example for all simulated datasets. This ignores the uncertainty in the within‐study variance components and we return to this issue below.

A thousand simulated datasets were produced for each combination of $\tau_{\beta }^{2}$ and $\tau_{\omega }^{2}$ so that in total 15 000 simulated network meta‐analyses were generated. All three estimation methods were applied to the same simulated datasets. In just 4 of these 15 000 simulated datasets the *metafor* package failed to provide results using REML with the defaults of *rma.mv*. This is due to the difficulties associated with the numerical methods used, rather than the REML estimator itself. This is a remarkably small proportion given that REML requires the numerical maximisation of the restricted log likelihood that depends on 2 unknown parameters. The results of these 4 datasets were discarded when calculating the repeated sampling properties of REML, and their impact on the overall results will in any case be negligible. In practice, however, a skilled statistician would be likely to change the defaults and so force convergence and/or investigate the reasons for this in real applications. Three of these 4 datasets occurred when $\tau_{\beta }^{2}=\tau_{\omega }^{2}=0$, which suggests that it may be more difficult to estimate excess variances when no such additional variation is present.

For all 15 parameter combinations, we calculated the mean estimate of $\tau_{\beta }^{2}$ and $\tau_{\omega }^{2}$, and the empirical standard deviation of these estimates. We also calculated the correlations between pairs of these estimates, with the conjecture that this might be highest between the Paule and Mandel and REML estimators because these were in better numerical agreement in the real example. Using correlations to determine the association between variables that are truncated at zero, as is the case here (this often occurs when the unknown variance components are 0 or small) is not ideal but these correlations were still thought to have the potential to determine which of the three estimation methods agree most closely. We use the ordered triple *ρ*_*β*_ to denote the estimated correlation between the Paule and Mandel and the DerSimonian and Laird ${\hat{\tau }}_{\beta }^{2}$, the Paule and Mandel and the REML ${\hat{\tau }}_{\beta }^{2}$, and the DerSimonian and Laird and the REML ${\hat{\tau }}_{\beta }^{2}$, in that order. The ordered triple *ρ*_*ω*_ contains these same correlations for the ${\hat{\tau }}_{\omega }^{2}$.

It is also of interest to know if better estimation of unknown variance components leads to better inference for the treatment effects, and so the coverage probability of the nominal 95% confidence intervals for basic parameters (across all 8 basic parameters) was also calculated. Recall that the inference for the treatment effects proceeds as a weighted regression where all weights are treated as known (but $\tau_{\beta }^{2}$ and $\tau_{\omega }^{2}$ are estimated) so that it was anticipated that the actual coverage probabilities would deviate from the nominal 95% coverage probability and in general would be slightly less than this.

### 6.2 Simulation study results

The results are shown in Table 3. With a few exceptions, the Paule and Mandel estimates of $\tau_{\beta }^{2}$ and $\tau_{\omega }^{2}$ appear to exhibit a little positive bias (where the average estimated value is greater than the true value), as expected because any estimates that would otherwise be negative are truncated at 0. The exceptions to this are for the estimates of $\tau_{\omega }^{2}$ when $\tau_{\beta }^{2}=0$, which are negatively biased. The Paule and Mandel estimates of $\tau_{\beta }^{2}$ appear to be the most positively biased when $\tau_{\beta }^{2}=0$, which explains the smaller average ${\hat{\tau }}_{\omega }^{2}$ and the negative bias for the Paule and Mandel estimator of $\tau_{\omega }^{2}$ in this setting. Recall that we substitute the estimate of $\tau_{\beta }^{2}$ into the non‐linear equation Equation 15 when calculating ${\hat{\tau }}_{\omega }^{2}$ so that an implication of using a positively biased estimate of $\tau_{\beta }^{2}$ is likely to be negative bias in ${\hat{\tau }}_{\omega }^{2}$. The proposed Paule and Mandel method therefore performs least favourably when $\tau_{\beta }^{2}=0$ but the resulting bias in ${\hat{\tau }}_{\omega }^{2}$ is not very severe. When $\tau_{\omega }^{2}=0$, so that the data are consistent, all methods perform satisfactorily. In general, the precision of the estimation depends on the estimation method used and in most settings the DerSimonian and Laird method has performed poorly in this respect. Overall, the proposed method performs well.

**Table 3:** Simulation study results

		PM			DL			REML				
$\tau_{\beta }^{2}$	$\tau_{\omega }^{2}$	${\hat{\tau }}_{\beta }^{2}$	${\hat{\tau }}_{\omega }^{2}$	CP	${\hat{\tau }}_{\beta }^{2}$	${\hat{\tau }}_{\omega }^{2}$	CP	${\hat{\tau }}_{\beta }^{2}$	${\hat{\tau }}_{\omega }^{2}$	CP	*ρ*_*β*_	*ρ*_*ω*_
0	0	0.03 (0.05)	0.01 (0.06)	0.97	0.01 (0.01)	0.01 (0.01)	0.97	0.00 (0.00)	0.00 (0.01)	0.96	(0.94, 0.11, 0.24)	(0.16, 0.27, 0.10)
0	0.25	0.03 (0.05)	0.20 (0.18)	0.88	0.01 (0.01)	0.26 (0.29)	0.89	0.00 (0.02)	0.26 (0.17)	0.91	(0.92, 0.50, 0.57)	(0.25, 0.77, 0.52)
0	0.50	0.03 (0.05)	0.40 (0.28)	0.88	0.01 (0.01)	0.51 (0.55)	0.87	0.00 (0.02)	0.50 (0.29)	0.91	(0.93, 0.47, 0.54)	(0.26, 0.82, 0.47)
0.25	0	0.26 (0.15)	0.08 (0.14)	0.96	0.26 (0.26)	0.11 (0.21)	0.94	0.23 (0.12)	0.05 (0.10)	0.95	(0.30, 0.84, 0.45)	(0.23, 0.77, 0.37)
0.25	0.25	0.27 (0.15)	0.27 (0.27)	0.92	0.25 (0.25)	0.30 (0.44)	0.88	0.25 (0.13)	0.25 (0.24)	0.92	(0.29, 0.85, 0.44)	(0.28, 0.88, 0.39)
0.25	0.50	0.26 (0.16)	0.52 (0.41)	0.91	0.25 (0.25)	0.57 (0.77)	0.87	0.25 (0.14)	0.51 (0.38)	0.91	(0.30, 0.88, 0.45)	(0.28, 0.93, 0.37)
0.50	0	0.51 (0.24)	0.12 (0.20)	0.96	0.52 (0.52)	0.22 (0.43)	0.95	0.46 (0.19)	0.08 (0.15)	0.96	(0.30, 0.87, 0.40)	(0.20, 0.82, 0.25)
0.50	0.25	0.51 (0.23)	0.32 (0.35)	0.93	0.50 (0.49)	0.44 (0.73)	0.90	0.48 (0.20)	0.28 (0.31)	0.92	(0.33, 0.90, 0.41)	(0.27, 0.89, 0.34)
0.50	0.50	0.52 (0.24)	0.56 (0.48)	0.92	0.50 (0.51)	0.64 (1.00)	0.88	0.50 (0.21)	0.54 (0.45)	0.92	(0.31, 0.92, 0.38)	(0.27, 0.94, 0.32)
0.75	0	0.75 (0.30)	0.17 (0.27)	0.96	0.72 (0.72)	0.33 (0.66)	0.94	0.69 (0.24)	0.13 (0.23)	0.95	(0.33, 0.90, 0.39)	(0.33, 0.85, 0.39)
0.75	0.25	0.76 (0.31)	0.35 (0.43)	0.93	0.72 (0.77)	0.53 (0.89)	0.90	0.72 (0.27)	0.33 (0.39)	0.92	(0.31, 0.92, 0.37)	(0.26, 0.91, 0.30)
0.75	0.50	0.75 (0.31)	0.59 (0.55)	0.93	0.72 (0.76)	0.83 (1.28)	0.89	0.73 (0.27)	0.56 (0.52)	0.93	(0.28, 0.93, 0.34)	(0.26, 0.93, 0.30)
1.00	0	1.02 (0.38)	0.20 (0.34)	0.95	1.00 (0.98)	0.45 (0.89)	0.94	0.95 (0.31)	0.15 (0.27)	0.95	(0.29, 0.90, 0.35)	(0.18, 0.85, 0.19)
1.00	0.25	1.01 (0.39)	0.38 (0.48)	0.94	1.01 (1.01)	0.63 (1.13)	0.91	0.96 (0.34)	0.33 (0.43)	0.93	(0.24, 0.93, 0.31)	(0.33, 0.90, 0.34)
1.00	0.50	1.01 (0.41)	0.57 (0.61)	0.92	1.03 (1.01)	0.85 (1.39)	0.89	0.98 (0.36)	0.53 (0.58)	0.92	(0.31, 0.94, 0.38)	(0.32, 0.93, 0.33)

One thousand simulated datasets were produced for each run so that 15 000 simulated datasets were produced in total. We show the mean ${\hat{\tau }}_{\beta }^{2}$ and ${\hat{\tau }}_{\omega }^{2}$ for all 3 estimation methods Empirical standard deviations are shown in parentheses. We also show the proportion of nominal 95% confidence intervals for the basic parameters that contain the true values (the estimated coverage probability, “CP”). Finally, we show the correlations between the 3 sets of estimates of $\tau_{\beta }^{2}$ and $\tau_{\omega }^{2}$ as the ordered triples *ρ*_*β*_ and *ρ*_*ω*_.

Abbreviations: PM, the proposed Paule and Mandel estimation method; DL, a generalisation of DerSimonian and Laird's univariate method; REML: restricted maximum likelihood.

We simulate outcome data under the model (2) so that the truncation of the DerSimonian and Laird estimates of $\tau_{\beta }^{2}$ and $\tau_{\omega }^{2}$ results in positive bias.^14^ This bias is generally evident in Table 3 (the mean values of ${\hat{\tau }}_{\beta }^{2}$ of 0.72, when $\tau_{\beta }^{2}=0.75$, can be explained by Monte Carlo error). Although many of the results for the proposed Paule and Mandel and the DerSimonian and Laird method are similar, there are also some important differences. In particular, for all simulations where $\tau_{\beta }^{2}>0$, the empirical standard deviations of the Paule and Mandel estimates in Table 3 are smaller. The proposed Paule and Mandel method therefore provides more precise estimates of the unknown variance components than the DerSimonian and Laird method when between‐study heterogeneity is present. Furthermore, the Paule and Mandel method appears to help to reduce the positive bias in many of the DerSimonian and Laird estimates of $\tau_{\omega }^{2}$. Provided that between‐study heterogeneity is present, as is usually suspected to be the case, the proposed Paule and Mandel method appears to provide more accurate estimates of the unknown variance components. However, the DerSimonian and Laird method performs best when $\tau_{\beta }^{2}=0$ and so can also be expected to perform best when the between‐study heterogeneity is very small. Neither of these two estimators consistently outperforms the other, but on balance, it seems reasonable to assert that the Paule and Mandel estimator has outperformed the DerSimonian and Laird estimator.

However, REML can be seen to have performed best, because it generally provides the least biased and most precise estimates of $\tau_{\beta }^{2}$ and $\tau_{\omega }^{2}$. In comparison, the proposed Paule and Mandel provides viable a semiparametric alternative where convergence is assured even when using very simple numerical methods. Despite this, REML retains its position as the gold standard estimation method.

The correlations between the estimated variance components shown in Table 3 are generally strongest between the Paule and Mandel and REML estimators, and these correlations are around 0.9 when $\tau_{\beta }^{2}$ is large. This helps to explain why the Paule and Mandel and REML results for the heterogeneous real example are in better agreement than with those from the DerSimonian and Laird method. The only exception to this is that the correlations between the Paule and Mandel and DerSimonian and Laird estimates of $\tau_{\beta }^{2}$ are very high when $\tau_{\beta }^{2}=0$. Interpreting correlations in this setting is difficult when many estimators are truncated to 0, but this suggests that the Paule and Mandel estimator of $\tau_{\beta }^{2}$ agrees well with the DerSimonian and Laird estimator when $\tau_{\beta }^{2}$ is small but instead agrees well with the REML estimator when this parameter is larger.

Better estimation of the unknown variance components does seem to feed into more accurate inference concerning the basic parameters, but this effect is not very impressive. All methods are conservative (the estimated actual coverage probabilities are more than the nominal 95%) when $\tau_{\beta }^{2}=\tau_{\omega }^{2}=0$, as expected as we then unnecessarily include variance components in the model. However, the estimated REML coverage probabilities do not drop below 90% in any setting, whilst this happens twice for the Paule and Mandel method and 7 (out of 15) times for the DerSimonian and Laird method. This shows that making better inference for one aspect of a fitted model can have desirable outcomes for other aspects. This and other conclusions should however be interpreted with caution, as our findings may not generalise to other settings, and we discuss possibilities for further simulation studies below.

### 6.3 Possibilities for future simulation studies

There is a very wide range of possibilities to explore in future simulation studies. In particular, the simulation study described above does not allow for the uncertainty in the between‐study covariance structure. Future simulation studies could examine how the estimation methods perform when the assumed model is not true, for example, by allowing for the uncertainty in the standard deviations, and so the within‐study covariance structure, for continuous data. This type of issue becomes an even more pressing concern when applying methods that use normal approximations, such as ours, to noncontinuous data. In particular, future simulation studies could focus on the implications of using normal approximations when the outcome is binary, for example, when using log‐odds ratios or risk differences as the outcomes. Future work could also explore a variety of realistic distributions of study sizes and other important parameters. This is important because conclusions, such as those made above, might depend on the distribution of the study sizes or other aspects of the simulation study. If this is the case, then our conclusions are not likely to generalise to other settings.

We further suggest that the need for more simulation studies, and also large‐scale empirical investigations, is now pressing. This is because we now have three of the main univariate estimation methods successfully generalised to the network meta‐analysis setting. This simulation work should include scenarios where the model used for analysis is both true and an approximation. This is so that the implications of misspecifying the model can be assessed. Extensive simulation studies are likely to form the subject of further work and we encourage others to also consider this possibility. For now, the four real examples and our simulation study provide proof of concept that the proposed methodology performs well. Further simulation studies, and large‐scale empirical work, are however beyond the scope of the present paper.

## 7 DISCUSSION

We have developed a new estimation method for network meta‐analysis that extends the univariate Paule and Mandel estimator. The proposed method has been found to perform well in some examples, and also in a simulation study that was based on a new example. In particular, the proposed Paule‐Mandel estimator appears to outperform the also semi‐parametric DerSimonian and Laird method. However, the so‐called two‐step univariate DerSimonian and Laird estimators are possible,^38^ and it is also straightforward to extend the corresponding network DerSimonian and Laird method in this manner. This provides a potential way to improve the DerSimonian and Laird method to make it more attractive, and this may form the subject of future work. Good classical solutions for fitting models for network meta‐analysis now have the potential to have a huge impact in applied work. In situations where analysts are content to make normality assumptions for the unknown random‐effects, and there are no convergence problems, then REML would seem to be the best estimation method. We emphasise again that REML is the current gold standard. However, the proposed Paule‐Mandel method is likely to be the preferred approach if normality assumptions are to be explicitly avoided and, at the very least, provides a suitable sensitivity analysis when using likelihood based analyses as the primary analysis.

Extensions of the model and estimation methods are likely to be of interest. The model can easily be extended to include study level covariates, by including these in the design matrix **X** and in the location parameters ***δ*** in model (2). All three classical estimation methods can also be extended to include these covariates. Subsequent work will show how this can be achieved using DerSimonian and Laird type methods. The Paule and Mandel estimators that we propose here apply in regression models of this type, where the right hand sides of all estimating equations are replaced by the appropriate degrees of freedom. Another possible extension is to allow multiple outcomes as well as multiple treatment groups, in a type of analysis that might be referred to as a multivariate network meta‐analysis. Work in this area is ongoing but it is not obvious how well defined Paule and Mandel estimators might be obtained when there are multiple outcomes. This is because of the difficulties associated with ensuring that the point estimates are unique, and this is at best much more difficult in the context of using unstructured unknown covariance matrices that are typically used in multivariate meta‐analysis.

There is currently much interest in dose response models for network meta‐analysis, in situations where studies report outcomes for different treatment and dose combinations. Extending model (2) to this situation is therefore also likely to be of interest and simple extensions of our model of this type are obvious. For example, we could take **Y** to be the vector that is obtained by staking the estimates for all treatments and dose comparisons relative to their baseline treatment‐dose and modify **M**_1_ and **M**_2_ so that the random‐effects that are applied to the outcome data depend only on the treatment and design and not the dose. Next, we modify design matrices to describe the additional estimated treatment effects that are now stratified by dose, where these treatment effects initially do not depend on the dose level. Then the dose level effect may be included in the model by introducing further covariates, as explained in the previous paragraph. This is merely a suggestion and other closely related ideas are also possible. A difficulty with this approach is that by stratifying the outcome data by dose, the treatment groups may become too small for the normal approximations used in this paper to be acceptable.

In the univariate setting, confidence intervals for the between‐study variance using the Q profile method^31^, ^32^ naturally accompany Paule and Mandel estimators.^33^ Since all our pivots, like the Q profile pivot, are continuous and strictly decreasing in the variance to be estimated, these pivots can be used to provide analogous confidence intervals for one of the unknown variance parameters whilst treating the other variance as if known. This immediately leads to confidence intervals for $\tau_{\beta }^{2}$ under the consistency model. However, it is not so obvious how we might obtain confidence intervals for $\tau_{\beta }^{2}$ whilst allowing for the uncertainty in $\tau_{\omega }^{2}$, and vice versa, when using Q profile type methods. For now some form of bootstrapping provides a practical way to obtain confidence intervals for the unknown variance components when using either the Paule and Mandel or the DerSimonian and Laird estimation methods. As discussed by Jackson et al,^14^ our methods do not immediately result in estimates of the inconsistency parameters ***ω***_*d*_, rather their variance $\tau_{\omega }^{2}$ is estimated. This is analogous to estimating the between‐study variance in conventional univariate random‐effects meta‐analyses and not the individual true study effects. Empirical Bayes estimates of the inconsistency parameters could in principle be derived to identify where the inconsistencies in the network arise but we leave this as an avenue for further work. The use of prediction intervals when using classical models for network meta‐analysis with random inconsistency effects is another interesting possibility that also we leave as a possibility for future work.

Although we include inconsistency effects in our modelling, we recognise that there will be many instances where the consistency assumption provides a good description of the data. Furthermore, some analysts may wish to make this assumption, perhaps on the grounds that this is thought to be a necessary prerequisite for performing a network meta‐analysis. Where possible attempts should be made to explain and remove any inconsistences in the evidence base, severe inconsistencies should strongly discourage the use of network meta‐analysis because these are likely to make the results invalid unless the model is well identified and describes the data very well. Strategies for explaining and removing notable inconsistencies include performing subgroup and sensitivity analyses, and using adjusted treatment effects. However, we suggest that it will often be much more realistic to anticipate a little inconsistency and to include this in the model, and this is our proposal here.

We have assumed a relatively simple model for network meta‐analysis where there are just two unknown variances. More complicated models could be considered in network meta‐analyses where there is sufficient data to identify them, but we suggest that our modelling framework is more than adequate for most applications. However, it should be recognised that the successful generalisation of the univariate Paule and Mandel estimation method has relied upon the use of our simple model. Further methodological work would be needed to extend the Paule and Mandel estimation method to fit more complex models.

We have focussed on the estimation of the model here but other forms of inference are possible. In particular, probabilistic ranks and *I*^2^ statistics can be calculated in the way described by Jackson, et al.^14^ See their extensive discussion for other ideas for making further inferences when using semiparametric estimation methods to fit models of the type considered here.

To summarise, we have proposed a new estimation method for network meta‐analysis. This new method extends the univariate Paule and Mandel estimation method and has been found to perform well in a variety of examples and in a simulation study. We now have three classical estimation methods for network meta‐analysis models with random inconsistency effects. R computing code for the proposed method is provided in the supplementary materials, and we hope that this will serve to make our methods attractive to applied analysts.

## Supporting information

Data S1.
Supplementary information: Extending the Paule‐Mandel estimator to perform network meta‐analyses with random inconsistency effects

Data S2.
Estimates of basic parameters for the three examples

## PROVING THAT ALL 3 *Q* PIVOTS ARE STRICTLY DECREASING IN THE UNKNOWN VARIANCE

### 1

To provide a general proof, we introduce notation that can describe all 3 pivots in a flexible way. We will take *τ*^2^ to represent whichever unknown variance parameter is to be estimated. Hence, *τ*^2^ represents $\tau_{\beta }^{2}$ in Equations (10) and (12) and $\tau_{\omega }^{2}$ in Equation (14). We will use **Y** and $\hat{Y}$ to represent the outcome data and the fitted values in these equations so that **Y** and $\hat{Y}$ represent **Y**_*d*_ and ${\hat{Y}}_{d}$ in Equation (12). We use ***β*** to represent the regression parameters so that ***β*** represents ***δ*** in Equations (10) and (14) and represents ***β***_*d*_ in Equation (12). We prove that the design specific pivot in Equation (12) is strictly decreasing in $\tau_{\beta }^{2}$ so that this is also true for their sum, which is used for estimating $\tau_{\beta }^{2}$ under the full model.

We now emphasise the dependence of the estimated regression parameters $\hat{\beta }$ on *τ*^2^ by writing $\hat{\beta }={\left({\hat{\beta }}_{1}\left(\tau^{2}\right),{\hat{\beta }}_{2}\left(\tau^{2}\right),\cdots ,{\hat{\beta }}_{k}\left(\tau^{2}\right)\right)}^{T}$ where *k* is the length of ***β***, different variance structures result in different estimates of the regression parameters. The fitted vectors depend on *τ*^2^ through these estimated regression vectors and so we write now $\hat{Y}\left({\hat{\beta }}_{1}\left(\tau^{2}\right),{\hat{\beta }}_{2}\left(\tau^{2}\right),\cdots ,{\hat{\beta }}_{k}\left(\tau^{2}\right)\right)$ to emphasise the correct dependence structure.

When using the 3 pivots for estimation, the total precision matrices are of the form (*τ*^2^**M** + **C**)^−1^, where **C** is a constant matrix that is held fixed in the estimation and **M** denotes the matrix associated with *τ*^2^. In the estimating equation resulting from Equation (10), **C** = **S** and **M** = **M**_1_; in the estimating equation from Equation (12), **C** = **S**_*d*_ and **M** = **M**_*d*_; and in the estimating equation from Equation (14), $C=S+{\hat{\tau }}_{\beta }^{2}M_{1}$ and **M** = **M**_2_. Although Equation (14) is a function of both variances, when estimating $\tau_{\omega }^{2}$, we use this pivot with fixed $\tau_{\beta }^{2}={\hat{\tau }}_{\beta }^{2}$ as explained above.

Using this flexible notation, we can represent all 3 pivots as used for estimation as

(A1) $$\begin{array}{l} Q\left(\tau^{2},{\hat{\beta }}_{1}\left(\tau^{2}\right),{\hat{\beta }}_{2}\left(\tau^{2}\right),\cdots ,{\hat{\beta }}_{k}\left(\tau^{2}\right)\right)\\ ={\left(Y-\hat{Y}\left({\hat{\beta }}_{1}\left(\tau^{2}\right),{\hat{\beta }}_{2}\left(\tau^{2}\right),\cdots ,{\hat{\beta }}_{k}\left(\tau^{2}\right)\right)\right)}^{T}{\left(\tau^{2}M+C\right)}^{-1}\left(Y-\hat{Y}\left({\hat{\beta }}_{1}\left(\tau^{2}\right),{\hat{\beta }}_{2}\left(\tau^{2}\right),\cdots ,{\hat{\beta }}_{k}\left(\tau^{2}\right)\right)\right) \end{array}$$

The chain rule for multivariate calculus gives

(A2) $$\frac{\partial Q}{\partial \tau^{2}}=R^{T}\left\{\frac{\partial }{\partial \tau^{2}\left[{\left(\tau^{2}M+C\right)}^{-1}\right]}\right\}R+\sum_{i=1}^{k}‍\left\{\frac{\frac{\partial Q}{\partial {\hat{\beta }}_{i}\}\{\partial {\hat{\beta }}_{i}}}{\partial \tau^{2}}\right\}$$

where $R=Y-\hat{Y}\left({\hat{\beta }}_{1}\left(\tau^{2}\right),{\hat{\beta }}_{2}\left(\tau^{2}\right),\cdots ,{\hat{\beta }}_{k}\left(\tau^{2}\right)\right)$ denotes the residuals.

Next, following Jackson et al.^33^, consider the estimates $\hat{\beta }={\left({\hat{\beta }}_{1},{\hat{\beta }}_{2},\cdots ,{\hat{\beta }}_{k}\right)}^{T}$ for a fixed value of *τ*^2^ using maximum likelihood estimation (or equivalently weighted least squares) under the normal model for **Y** with covariance matrix *τ*^2^**M** + **C**. The log‐likelihood is proportional to

$$\ell \left(\beta_{1},\beta_{2},\cdots ,\beta_{k}\right)={\left(Y-\hat{Y}\left(\beta_{1},\beta_{2},\cdots ,\beta_{k}\right)\right)}^{T}{\left(\tau^{2}M+C\right)}^{-1}\left(Y-\hat{Y}\left(\beta_{1},\beta_{2},\cdots ,\beta_{k}\right)\right),$$

where *τ*^2^ is held fixed. This likelihood is of the same form as Equation A1, and we solve $\text{∂}\ell /\text{∂}{\hat{\beta }}_{i}=\text{∂}Q/\text{∂}{\hat{\beta }}_{i}=0$ to estimate the regression parameters and so calculate the fitted values in the quadratic form A1. The summation in Equation (A2) is therefore 0 (because $\text{∂}Q/\text{∂}{\hat{\beta }}_{i}=0$ for all *i* = 1 , 2 ,  ⋯ *k*) and the derivative drastically simplifies.

Next, we require a standard result relating to matrix differentiation. If **F** is a matrix whose entries are functions of **x** = (*x*_1_, ⋯*x*_*m*_)^*T*^, then the matrix that contains the partially differentiated entries of **F** with respect to *x*_*j*_ is denoted as ∂**F**/∂*x*_*j*_ (Harville p.296, 39). Then the required result is expression (8.15) of Harville (page 311) which, assuming **F** is square and nonsingular, is

(A3) $$\frac{\partial F^{-1}}{\partial x_{j}}=-F^{-1}\left\{\frac{\partial F}{\partial x_{j}}\right\}F^{-1}$$

Taking **F** to be *τ*^2^**M** + **C** and *x*_*j*_ to be *τ*^2^ in Equation (A3), where ∂/∂*τ*^2^[*τ*^2^**M** + **C**] = **M**, we can evaluate Equation (A2) as

(A4) $$\partial Q/\partial \tau^{2}=-{\left(\Sigma^{-1}R\right)}^{T}M\left(\Sigma^{-1}R\right)$$

where **Σ** = *τ*^2^**M** + **C**. It was noted above that **M**_1_, **M**_2_, and **M**_*d*_ are symmetrical; hence, **M** is regardless of which of the 3 matrices **M** is taken to represent. **C** is also symmetrical in all 3 representations, because of this observation and also because the within‐study covariance matrix **S** is symmetrical. Hence, **Σ** = *τ*^2^**M** + **C** and its inverse are symmetrical, as required to present Equation (A4) in the form shown.

Finally, we have that **M** is positive semidefinite, because it is proportional to the covariance matrix for the unknown random‐effects. One definition of a positive definite matrix *A* is that **y**^*T*^**Ay** > 0 for all vectors **y**. Then taking **A** = **M** and **x** = **Σ**^−1^**R** in this definition of positive definiteness immediately results in the conclusion that

(A5) $$\partial Q/\partial \tau^{2}=-{\left(\Sigma^{-1}R\right)}^{T}M\left(\Sigma^{-1}R\right)<0$$

so that all 3 pivots used for estimation are strictly decreasing in the variance parameters that they estimate. The proposed Paule and Mandel estimators are therefore unique. Equation (A5) is more general than the result given by Jackson et al^33^ for univariate meta‐regression, and this previous result is recovered by taking **M** to be the identity matrix and **Σ** to be a diagonal matrix containing the total variances of each study; in a univariate meta‐regression, all estimates are independent. This derivative has been checked numerically and could be used in Newton‐Raphson numerical methods to solve estimating equations (Equations (11), (13), and (15)) in the way explained by Jackson et al,^33^ but in practice, it is more straightforward to use simple root finding methods such as bisection.

## References

[1] Salanti G . Indirect and mixed‐treatment comparison, network, or multiple‐treatments meta‐analysis: many names, many benefits, many concerns for the next generation evidence synthesis tool. Research Synthesis Methods. 2012;3:80‐97. 26062083 10.1002/jrsm.1037

[2] Salanti G , Higgins JPT , Ades AE , Ioannidis JPA . Evaluation of networks of randomized trials. Statical Methods in Medical Research. 2008;17:279‐301. 10.1177/0962280207080643 17925316

[3] Ades AE , Lu G , Higgins JP . The interpretation of random‐effects meta‐analysis in decision models. Med Decis Making. 2005;25:646‐654. 16282215 10.1177/0272989X05282643

[4] DerSimonian R , Laird N . Meta‐analysis in clinical trials. Control Clin Trials. 1986;7:177‐188. 3802833 10.1016/0197-2456(86)90046-2

[5] Hardy RJ , Thompson SG . A likelihood approach to meta‐analysis with random effects. Stat Med. 1996;15:619‐629. 8731004 10.1002/(SICI)1097-0258(19960330)15:6<619::AID-SIM188>3.0.CO;2-A

[6] Higgins JPT , Thompson SG , Spiegelhalter DJ . A re‐evaluation of random‐effects meta‐analysis. Journal of the Royal Statistical Society, Series A. 2009;172:137‐159. 10.1111/j.1467-985X.2008.00552.x PMC2667312 19381330

[7] Lu G , Ades AE . Combination of direct and indirect evidence in mixed treatment comparisons. Stat Med. 2004;23:3105‐3124. 15449338 10.1002/sim.1875

[8] Higgins JPT , Jackson D , Barrett JK , Lu G , Ades AE , White IR . Consistency and inconsistency in network meta‐analysis: concepts and models for multi‐arm studies. Research Synthesis Methods. 2012;3:98‐110. 26062084 10.1002/jrsm.1044 PMC4433772

[9] White IR , Barrett JK , Jackson D , Higgins JPT . Consistency and inconsistency in network meta‐analysis: model estimation using multivariate meta‐regression. Research Synthesis Methods. 2012;3:111‐125. 26062085 10.1002/jrsm.1045 PMC4433771

[10] Piepho HP . Network meta‐analysis made easy: detection of inconsistency using factorial analysis‐of‐variance models. BMC Med Res Methodol. 2014;14:61. 24885590 10.1186/1471-2288-14-61 PMC4049370

[11] Piepho HP , Williams ER , Madden LV . The use of two‐way linear mixed models in multitreatment meta‐analysis. Biometrics. 2012;68:1269‐1277. 22845838 10.1111/j.1541-0420.2012.01786.x

[12] Jackson D , Boddington P , White IR . The design‐by‐treatment interaction model: a unifying framework for modelling loop inconsistency in network meta‐analysis. Research Synthesis Methods. 2016;7:329‐332. 26588593 10.1002/jrsm.1188 PMC4946625

[13] Jackson D , Barrett JK , Rice S , White IR , Higgins JPT . A design‐by‐treatment interaction model for network meta‐analysis with random inconsistency effects. Stat Med. 2014;33:3639‐3654. 24777711 10.1002/sim.6188 PMC4285290

[14] Jackson D , Law M , Barrett JK , et al. Extending DerSimonian and Laird's methodology to perform network meta‐analyses with random inconsistency effects. Stat Med. 2016;35:819‐839. 26423209 10.1002/sim.6752 PMC4973704

[15] Law M , Jackson D , Turner R , Rhodes K , Viechtbauer W . Two new methods to fit models for network meta‐analysis with random inconsistency effects. BMC Med Res Methodol. 2016;16:87. 27465416 10.1186/s12874-016-0184-5 PMC4964019

[16] Paule RC , Mandel J . Concensus values and weighting factors. J Res Natl Bur Stand. 1982;87:377‐385. 10.6028/jres.087.022 PMC6768160 34566088

[17] Veroniki AA , Jackson D , Viechtbauer W , et al. Methods to estimate the between‐study variance and its uncertainty in meta‐analysis. Research Synthesis Methods. 2016;7:55‐79. 26332144 10.1002/jrsm.1164 PMC4950030

[18] Hoaglin DC . Misunderstandings about Q and ‘Cochran's Q test’ in meta‐analysis. Stat Med. 2016;35:485‐495. 26303773 10.1002/sim.6632

[19] Kulinskaya E , Dollinger MB , Bjørkestøl K . On the moments of Cochran's Q statistic under the null hypothesis, with application to the meta‐analysis of risk difference. Research Synthesis Methods. 2011;2:254‐270. 26061889 10.1002/jrsm.54

[20] Rukhin AL , Biggerstaff BJ , Vangel MG . Restricted maximum likelihood estimation of a common mean and the Mandel‐Paule algorithm. Journal of Statistical Planning and Inference. 2010;83:319‐330.

[21] Viechtbauer W , Lopez‐Lopez JA , Sanchez‐Meca J , Marin‐MartÃ‐nez F . A comparison of procedures to test for moderators in mixed‐effects meta‐regression models. Psychol Methods. 2014;20:360‐374. 25110905 10.1037/met0000023

[22] Kontopantelis E , Reeves D . Performance of statistical methods for meta‐analysis when true study effects are non‐normally distributed: a simulation study. Stat Methods Med Res. 2012a;21:409‐426. 21148194 10.1177/0962280210392008

[23] Kontopantelis E , Reeves D . Performance of statistical methods for meta‐analysis when true study effects are non‐normally distributed: a comparison between DerSimonian Laird and restricted maximum likelihood. Stat Methods Med Res. 2012b;21:657‐659. 23171971 10.1177/0962280211413451

[24] Dias S , Ades AE . Absolute or relative effects? Arm‐based synthesis of trial data. Research Synthesis Methods. 2016;7:23‐28. 26461457 10.1002/jrsm.1184 PMC5102631

[25] Hong H , Chu H , Zhang J , Carlin BP . Rejoinder to the discussion of “a Bayesian missing data framework for generalized multiple outcome mixed treatment comparisons,” by S. Dias and A.E. Ades. Research Synthesis Methods. 2016;7:29‐33. 26461816 10.1002/jrsm.1186 PMC4779393

[26] Gasparrini A , Armstrong B , Kenward MG . Multivariate meta‐analysis for non‐linear and other multi‐parameter associations. Stat Med. 2012;7:3821‐3839. 10.1002/sim.5471 PMC3546395 22807043

[27] Jackson D , White IR , Riley RD . Multivariate meta‐analysis: potential and promise. Stat Med. 2012;30:2481‐2498. 10.1002/sim.4172 PMC3470931 21268052

[28] Krahn U , Binder H , König J . A graphical tool for locating inconsistency in network meta‐analyses. BMC Research Methodology. 2013;13:35. 10.1186/1471-2288-13-35 PMC3644268 23496991

[29] Viechtbauer W . Conducting meta‐analyses in R with the metafor package. Journal of Statistical Software. 2010;36:1‐48.

[30] Davey J , Turner RM , Clarke MJ , Higgins JPT . Characteristics of meta‐analyses and their component studies in the Cochrane database of systematic reviews: a cross‐subsectional, descriptive analysis. BMC Med Res Methodol. 2011;11:160. 22114982 10.1186/1471-2288-11-160 PMC3247075

[31] Knapp G , Biggerstaff BJ , Hartung J . Assessing the amount of heterogeneity in random‐effects meta‐analysis. Biom J. 2006;48:271‐285. 16708778 10.1002/bimj.200510175

[32] Viechtbauer W . Confidence intervals for the amount of heterogeneity in a meta‐analysis. Stat Med. 2007;26:37‐52. 16463355 10.1002/sim.2514

[33] Jackson D , Turner R , Rhodes K , Viechtbauer W . Methods for calculating confidence and credible intervals for the residual between‐study variance in random effects meta‐regression models. BMC Med Res Methodol. 2014;14:103 25196829 10.1186/1471-2288-14-103 PMC4160560

[34] Searle SR . Linear Models. Chichester: Wiley; 1971.

[35] Tricco A et al. Comparative effectiveness of cognitive enhancers for treating Alzheimer's dementia: a systematic review and network meta‐analysis. BMJ. 2016; Under review.

[36] Rücker G , Schwarzer G 2013 netmeta: Network meta‐analysis with R. *R package version 0.9‐2*.

[37] Higgins JPT , Thompson SG . Quantifying heterogeneity in a meta‐analysis. Stat Med. 2002;21:1539‐1558. 12111919 10.1002/sim.1186

[38] DerSimonian R , Kacker R . Random‐effects model for meta‐analysis of clinical trials: an update. Clin Trials. 2007;28:105‐114. 10.1016/j.cct.2006.04.004 16807131

[39] Harville DA . Matrix Algebra from a Statistician's Perspective. New York: Springer; 2012.

