# Supplementary material for: Paule‐Mandel estimators for network meta‐analysis with random inconsistency effects
Source: Res Synth Methods. 2017 Jun 5;8(4):416–34. doi: 10.1002/jrsm.1244 (PMC5720360; doi:10.1002/jrsm.1244)
Supplement: Supplementary file 1 — Data S1. Supplementary information: Extending the Paule‐Mandel estimator to perform network meta‐analyses with random inconsistency effects [file JRSM-8-416-s001.docx]

**Supplementary information: Extending the Paule-Mandel estimator to perform network meta-analyses with random inconsistency effects**

**R function for all applications**

The function takes a single argument, a list. This list contains two further lists, y and s, and the vector d, all of length equal to the total number of studies. The list y contains the estimated treatment effects *y_d_*_i_, which are scalars if there is only one effect (e.g. design *BC*), vectors otherwise. The list s contains the within-study covariance matrices *s_di_*, which again may be scalars if there is only one effect. The vector d contains the design of each study, (e.g. *AB*, *AC*, *BDE*). Each treatment should be allocated a letter, with *A* being the reference treatment. The notation closely follows that of the main paper.

*Designs need not be specified in alphabetical order, so that for example the design EDC is allowed. However designs should be specified in the same way throughout. For example, EDC and CDE would be treated as if two different designs. All entries of the within-study covariance matrices should be positive (the non-main diagonal entries are variances of the average outcome in the baseline arm) and this has been assumed throughout.*

The main results included in the output are: est, the estimated treatment effects; Cov, the covariance matrix for the active treatments; se, the standard error of each estimated treatment effect; p, the associated p values; tau2beta, tau2omega, the (*untruncated*) estimates of the variance components $\tau_{\beta}^{2}$ and $\tau_{\omega}^{2}$ respectively. The truncated estimates can of course be obtained from their untruncated counterparts.

Corresponding estimates are also obtained under the assumption of consistency (i.e. $\tau_{\omega}^{2}$ is assumed to equal zero – the "random-effects and consistent" model); these results are appended with “_con”. Finally, the *I^2^* statistics are returned: *I_12_^2^* compares the random-effects and consistent model with the “common-effect and consistent” model; *I_13_^2^* compares the main random-effects and inconsistent model with the common-effect and consistent model, and *I_23_^2^* compares the main random-effects and inconsistent model with the random-effects and consistent model.

*The Matrix package is required for the program to run.*

Cut and paste the code below into R to produce the main results for the OAK data.

fn_pm<-function(all_data, ub=100){

require(Matrix) # Necessary for bdiag function

n <- length(unlist(all_data$y)) # Total number of treatment effects

smat=bdiag(all_data$s) # Create and fill matrix S

############## Create and fill matrix X ##############

trts <- 0 # Number of treatments, including placebo

for(i in 1:26) trts <- trts + grepl(LETTERS[i], paste(all_data$d,collapse=""))

X=NULL

for(i in 1:length(all_data$d))

{ # For each study d, create a small matrix [no. active trts in study i] x [total no. trts-1].

des_letters=strsplit(all_data$d[i], "")[[1]]

des_numbers=match(des_letters, LETTERS)

cons=length(des_numbers)-1

X1=matrix(0, nrow=cons, ncol=trts) # Create the empty X1 matrix;

for(j in 1:cons) X1[j, des_numbers[j+1]]=1 # Add 1's in the columns of active treatments;

X1[,des_numbers[1]]=-1 # Add a -1 in the column of the (relative) reference trt;

X1 <- X1[, -1]

X=rbind(X, X1, deparse.level = 0) # Add small X1 matrices to create full design matrix X

}

############## Create matrix P1 ##############

p1 <- smat # Same zero terms as S, but

p1[which(p1[,]!=0)] <- 0.5 # all non-zero terms should equal 0.5, except

diag(p1) <- 1 # the diagonal, which should equal 1

############# Create matrix P2 ################

designs=NULL

for(i in 1:length(all_data$y))

{

new_designs=rep(all_data$d[i], length(all_data$y[[i]]))

designs=c(designs, new_designs)

}

p2 <- matrix(0, nrow=n, ncol=n)

for(i in 1:n)

for(j in 1:n)

{

if(designs[i]==designs[j] & sum(X[i,]==X[j,])==trts-1) p2[i,j]=1

if(designs[i]==designs[j] & sum(X[i,]==X[j,])!=trts-1) p2[i,j]=0.5

}

### New code to implement Paule-Mandel estimation

### This new code requires functions Q and Qd, that calculate the quadratic forms required.

### The "weights" in these quadratic forms depend on the unknown variance to be estimated.

### We solve these quadratic forms as equal to their dof to provide estimates.

### Estimate between-study heterogeneity

check=Qd(0, all_data, designs)

if (check<=0) tau2beta=0

if (check>0) tau2beta=uniroot(Qd, all_data=all_data, designs=designs, lower=0, upper=ub)$root

### Estimate between-study heterogeneity under the consistency assumption

check_con=Q(0, all_data, p1, smat, X,trts)

if (check_con<=0) tau2beta_con=0

if (check_con>0) tau2beta_con=uniroot(Q, all_data=all_data, P_mat=p1, Fixed_mat=smat, X=X, trts=trts, lower=0, upper=ub)$root

### Estimate the inconsistency variance

check_incon=Q(0, all_data, p2, smat+tau2beta*p1, X, trts)

if (check_incon<=0) tau2omega=0

if (check_incon>0) tau2omega=uniroot(Q, all_data=all_data, P_mat=p2, Fixed_mat=smat+tau2beta*p1, X=X, trts=trts, lower=0, upper=ub)$root

### End of new code to implement Paule-Mandel estimation

y <- unlist(all_data$y)

v <- smat + tau2beta*p1 + tau2omega*p2

v_inv <- solve(v)

Cov <- solve(t(X) %*% v_inv %*% X)

est <- Cov %*% t(X) %*% v_inv %*% y

Vcon = smat + tau2beta_con*p1

WWcon=solve(Vcon)

Cov_con=solve(t(X) %*% WWcon %*% X)

est_con=Cov_con %*% t(X) %*% WWcon %*% y

sd=diag(Cov)^0.5;

sd_con=diag(Cov_con)^0.5

p=pnorm(-abs(as.numeric(est))/sd)*2 ;

p_con=pnorm(-abs(as.numeric(est_con))/sd_con)*2

w <- solve(smat)

# Fit model under "common-effect and consistent" (CC) model (i.e. assuming tau2_beta and tau2_omega are zero):

Cov_CC_inv <- t(X) %*% w %*% X

Cov_CC <- solve(Cov_CC_inv)

power=1/(2*(trts-1))

# R and I statistics comparing RC model and CC model:

R12=det(Cov_con %*% Cov_CC_inv)^power

I_RC_CC=(R12^2-1)/R12^2

# R and I statistics comparing "random-effects and inconsistent" (RI) model and CC model:

R13=det(Cov%*%(Cov_CC_inv))^power

I_RI_CC=(R13^2-1)/R13^2

# R and I statistics comparing RI model and RC model:

R23=det(Cov%*%(solve(Cov_con)))^power

I_RI_RC=(R23^2-1)/R23^2

return(list(est=as.matrix(est), Cov=as.matrix(Cov), se=sd , p=p, tau2beta=tau2beta, tau2omega=tau2omega,

est_con=as.matrix(est_con), Cov_con=as.matrix(Cov_con),se_con=sd_con, p_con=p_con,

tau2beta_con=tau2beta_con, I12=I_RC_CC, I13=I_RI_CC, I23=I_RI_RC))

}

Q<-function (x, all_data, P_mat, Fixed_mat, X, trts)

{

y <- unlist(all_data$y)

n <- length(y)

dof <- n-trts+1

w <- solve(Fixed_mat+x*P_mat)

b <- w - w %*% X %*% solve(t(X) %*% w %*% X) %*% t(X) %*% w

qnet <- as.numeric(t(y) %*% b %*% y)

qnet-dof

}

Qd<-function (x, all_data, designs)

{

smat=as.matrix(bdiag(all_data$s)) # Create and fill matrix S

################# Find Qhet_d, where #################

# Qhet_d = Y_d' B_d Y_d

#dof = n-trts+1 # Degrees of freedom

unique_d <- unique(all_data$d) # Each unique design

d <- length(unique_d) # Number of unique studies

q_list <- list(qhet=NULL, dof=NULL, k=NULL, ndcd=NULL)

for(i in 1:d){ # For design i, record:

nd <- sum(unique_d[i]==all_data$d) # Number of studies

which_d <- which(unique_d[i]==all_data$d) # Which studies have design i?

yd <- unlist(all_data$y[which_d]) # Treatment effects assoc'd with studies of design i

cd <- nchar(unique_d[i])-1 # No. of treatments compared to reference treatment

icd <- diag(cd) # Identity matrix Icd

xd <- matrix(rep(icd, nd), ncol=cd, byrow=T) # Stacked identity matrices

sd <- smat[which(unique_d[i]==designs), which(unique_d[i]==designs)] # Block diag. matrix containing S_di matrices

pcd <- matrix(rep(0.5, cd*cd), nrow=cd)

diag(pcd) <- 1

ind <- diag(nd) # Identity matrix with dim=number of studies

kron <- ind %x% pcd # Kronecker product I_nd (x) P_cd

sd<-sd+x*kron # sd is now Total variance

wd <- solve(sd)

bd <- wd - wd %*% xd %*% solve(t(xd) %*% wd %*% xd) %*% t(xd) %*% wd

kd <- sum(diag(bd %*% kron))

q_list$qhet[i] <- as.numeric(t(yd) %*% bd %*% yd)

q_list$dof[i] <- (nd-1)*cd

q_list$k[i] <- kd

q_list$ndcd[i] <- nd*cd

}

################## Find tau2_beta #####################

qhet <- sum(q_list$qhet)

dof <- sum(q_list$dof)

qhet-dof

}

#Alzheimer’s data

ALZ=structure(list(y = list(0.3, 0.68, -0.4, 1.7, 1, 1.7, 0.2, 0.83,

-0.95, 0.65, 0.3, 0.7, 0.96, -1.4, 0.6, 1.06, 0.49, c(0.8,

1.1), 1, 1.6, 4.83, 1.35, 1.57, -0.4, 4.21, 0.73, 1.4, 0.9,

-0.7, 0.3, 0.11, c(0.41, 0.370000000000001, 0.82), c(-2.4,

-0.199999999999999), c(-0.0599999999999987, -0.120000000000001

), 0.300000000000001, -0.31, 1.77, -1, 0.7, 1.36, 1.5), s = list(

0.00410558459878848, 0.151213716312057, 0.260384362934363,

0.0017720704845815, 0.15671772615628, 0.609905144124169,

1.47862904392765, 0.210880938416422, 0.180401785714286, 0.848642307692308,

0.00181589576652739, 0.530536290322581, 0.120116307692308,

0.320736043956044, 0.290033247667999, 0.328408800773694,

0.464275333333333, structure(c(0.0108896797153025, 0.000889679715302491,

0.000889679715302491, 0.00177327971530249), .Dim = c(2L,

2L)), 0.180401666666667, 2.73732727272727, 0.2813, 0.23284064171123,

3.14928571428571, 0.244942222222222, 1.29641025641026, 0.0105873468989411,

0.386736020923521, 0.212621191646192, 0.643788961038961,

0.266223478835979, 0.217399061522419, structure(c(0.649618181818182,

0.293254545454545, 0.293254545454545, 0.293254545454545,

0.517272727272727, 0.293254545454545, 0.293254545454545,

0.293254545454545, 0.509272727272727), .Dim = c(3L, 3L)),

structure(c(1.69368421052632, 0.973157894736842, 0.973157894736842,

1.73551083591331), .Dim = c(2L, 2L)), structure(c(0.353215151515151,

0.20854696969697, 0.20854696969697, 0.38680303030303), .Dim = c(2L,

2L)), 0.729516010854817, 0.470418796992481, 0.212901785487355,

0.0202011904761905, 1.05254100547196, 0.162204329004329,

0.0798375128413085), d = c("AD", "AB", "EG", "AD", "AB",

"AB", "BF", "AB", "BC", "AC", "AE", "AF", "AB", "AB", "AB", "BC",

"BD", "ADE", "AB", "AF", "DE", "BH", "AB", "AB", "AH", "AC",

"AF", "AB", "AC", "AB", "AF", "FGHI", "BCE", "BCD", "AB", "AF",

"AB", "AD", "AB", "AB", "AB")), .Names = c("y", "s", "d"))

fn_pm(ALZ)
