# Supplementary material for: Paule‐Mandel estimators for network meta‐analysis with random inconsistency effects
Source: Res Synth Methods. 2017 Jun 5;8(4):416–34. doi: 10.1002/jrsm.1244 (PMC5720360; doi:10.1002/jrsm.1244)
Supplement: Supplementary file 2 — Data S2. Estimates of basic parameters for the three examples [file JRSM-8-416-s002.docx]

Estimates of basic parameters for the three examples

Example 1

| Basic parameter | PM | DL | REML |
| --- | --- | --- | --- |
| AB | -0.22 (0.11) | -0.22 (0.11) | -0.22 (0.11) |
| AC | -0.17 (0.28) | -0.17 (0.28) | -0.17 (0.28) |
| AD | -0.33 (0.32) | -0.33 (0.32) | -0.33 (0.32) |
| AE | -0.32 (0.25) | -0.32 (0.25) | -0.32 (0.25) |
| AF | -0.35 (0.28) | -0.35 (0.28) | -0.35 (0.28) |
| AG | -0.65 (0.83) | -0.65 (0.83) | -0.65 (0.83) |
| AH | -0.28 (0.47) | -0.28 (0.47) | -0.28 (0.47) |

Example 2

| Basic parameter | PM | DL | REML |
| --- | --- | --- | --- |
| AB | -2.01 (0.76) | -1.92 (0.64) | -1.97 (0.68) |
| AC | -1.42 (0.89) | -1.35 (0.74) | -1.40 (0.81) |
| AD | -0.74 (0.81) | -0.67 (0.67) | -0.66 (0.72) |

Example 3

| Basic parameter | PM | DL | REML |
| --- | --- | --- | --- |
| AB | 0.06 (0.26) | 0.04 (0.20) | 0.04 (0.20) |
| AC | 0.63 (0.39) | 0.60 (0.32) | 0.60 (0.32) |
| AD | −0.78 (0.20) | −0.78 (0.16) | −0.78 (0.16) |
| AE | −0.44 (0.32) | −0.46 (0.25) | −0.46 (0.25) |
| AF | −0.15 (0.62) | −0.15 (0.46) | −0.15 (0.46) |
| AG | −0.70 (0.29) | −0.59 (0.22) | −0.59 (0.22) |
| AH | −0.38 (0.14) | −0.37 (0.11) | −0.37 (0.11) |
| AI | −0.02 (0.38) | −0.03 (0.30) | −0.03 (0.30) |
| AJ | −0.03 (0.45) | −0.01 (0.35) | −0.01 (0.35) |
| AK | −0.28 (0.37) | −0.28 (0.29) | −0.28 (0.29) |
| AL | −0.38 (0.35) | −0.35 (0.26) | −0.35 (0.26) |
| AM | −0.24 (0.30) | −0.25 (0.23) | −0.25 (0.23) |
| AN | −0.25 (0.44) | −0.25 (0.37) | −0.25 (0.37) |
| AO | −1.11 (0.57) | −1.11 (0.48) | −1.11 (0.48) |
| AP | −0.24 (0.46) | −0.25 (0.36) | −0.25 (0.36) |
| AQ | −0.29 (0.38) | −0.29 (0.30) | −0.29 (0.30) |
| AR | 0.46 (0.69) | 0.46 (0.55) | 0.46 (0.55) |
| AS | −0.69 (0.41) | −0.70 (0.32) | −0.70 (0.32) |
| AT | 0.01 (0.41) | 0.01 (0.32) | 0.01 (0.32) |
| AU | −0.77 (0.72) | −0.78 (0.57) | −0.78 (0.57) |
| AV | −0.65 (0.27) | −0.63 (0.22) | −0.63 (0.22) |
